# Supplementary material for: DoubletDecon: Deconvoluting Doublets from Single-Cell RNA-Sequencing Data
Source: Cell Rep. Author manuscript; Available in PMC 2020 Jan 26. (PMC6983270; doi:10.1016/j.celrep.2019.09.082)
Supplement: 1 [file NIHMS1542667-supplement-1.pdf]

Cell Reports, Volume *29*

Supplemental Information

DoubletDecon: Deconvoluting Doublets

from Single-Cell RNA-Sequencing Data

Erica A.K. DePasquale, Daniel J. Schnell, Pieter-Jan Van Camp, Íñigo Valiente-Alandí, Burns C. Blaxall, H. Leighton Grimes, Harinder Singh, and Nathan Salomonis

Tool	Cell Count	ρ' (rank)	Minimum Unique	Sensitivity (Std. Dev.)	Specificity (Std. Dev.)
DoubletDecon	6,525	1.0 (1)	4†	42.66% (1.84%)	84.82% (1.47%)
DoubletDecon	6,525	1.0 (1)	5	44.73% (1.01%)	83.70% (1.07%)
DoubletDecon	6,525	1.0 (1)	6	45.40% (0.60%)	83.18% (0.38%)
DoubletDecon	6,525	1.0 (1)	10	45.52% (0.60%)	82.17% (0.52%)
DoubletDecon	6,525	1.1 (2)	4†	56.89% (1.45%)	80.98% (0.84%)
DoubletDecon	6,525	1.1 (2)	5	60.27% (0.58%)	79.98% (0.67%)
DoubletDecon	6,525	1.1 (2)	6	60.83% (0.90%)	79.31% (0.59%)
DoubletDecon	6,525	1.1 (2)	10	62.92% (0.59%)	77.49% (0.46%)
DoubletDecon	6,525	1.2 (3)	4†	57.45% (1.16%)	81.17% (0.59%)
DoubletDecon	6,525	1.2 (3)	5	60.39% (0.72%)	79.44% (0.66%)
DoubletDecon	6,525	1.2 (3)	6	60.46% (0.84%)	79.49% (0.57%)
DoubletDecon	6,525	1.2 (3)	10	62.24% (0.70%)	77.85% (0.81%)
DoubletDecon	14,619	1.1 (1)	4†	51.52% (3.10%)	83.58% (1.68%)
DoubletDecon	14,619	1.1 (1)	5	54.63% (2.20%)	82.09% (0.93%)
DoubletDecon	14,619	1.1 (1)	6	57.76% (1.02%)	80.98% (0.70%)
DoubletDecon	14,619	1.1 (1)	10	59.32% (0.33%)	79.80% (0.58%)
DoubletDecon	14,619	1.15 (2)	4†	51.11% (2.21%)	83.48% (0.88%)
DoubletDecon	14,619	1.15 (2)	5	55.37% (1.72%)	81.65% (1.15%)
DoubletDecon	14,619	1.15 (2)	6	57.40% (1.50%)	80.87% (0.91%)
DoubletDecon	14,619	1.15 (2)	10	59.35% (0.60%)	79.89% (0.52%)
DoubletDecon	14,619	1.2 (3)	4†	59.40% (1.95%)	78.02% (1.35%)
DoubletDecon	14,619	1.2 (3)	5	61.49% (1.93%)	76.61% (1.30%)
DoubletDecon	14,619	1.2 (3)	6	63.60% (1.27%)	75.41% (0.92%)
DoubletDecon	14,619	1.2 (3)	10	65.63% (0.60%)	73.92% (0.72%)

Tool	Cell Count	Rate	Homotypic Adjusted	Sensitivity (Std. Dev.)	Specificity (Std. Dev.)
DoubletFinder	6,525	10%	-	22.27% (3.05%)	93.44% (0.85%)
DoubletFinder	6,525	10%	7.6%	17.70% (2.62%)	95.28% (0.73%)
DoubletFinder	6,525	10.7%	-	23.44% (3.21%)	92.88% (0.89%)
DoubletFinder	6,525	10.7%	8.1%	18.84% (2.64%)	94.91% (0.73%)
DoubletFinder	6,525	12.5% (loading)†	-	26.44% (3.82%)	91.40% (1.07%)
DoubletFinder	6,525	12.5% (loading)†	9.5%	21.31% (2.75%)	93.87% (0.76%)
DoubletFinder	6,525	15%	-	30.54% (3.99%)	89.35% (1.12%)
DoubletFinder	6,525	15%	11.3%	24.51% (3.11%)	92.34% (0.88%)
DoubletFinder	6,525	21.9% (true)	-	39.89% (5.17%)	83.12% (1.44%)
DoubletFinder	6,525	21.9% (true)	16.6%	32.87% (3.93%)	88.02% (1.09%)
DoubletFinder	14,619	10%	-	62.57% (1.69%)	96.30% (0.19%)
DoubletFinder	14,619	10%	7.6%	48.75% (1.17%)	97.56% (0.14%)
DoubletFinder	14,619	10.7% (true)	-	65.37% (1.72%)	95.85% (0.21%)
DoubletFinder	14,619	10.7% (true)	8.1%	51.94% (1.37%)	97.36% (0.17%)
DoubletFinder	14,619	12.5% (loading)†	-	72.14% (2.01%)	94.65% (0.25%)
DoubletFinder	14,619	12.5% (loading)†	9.5%	58.87% (1.58%)	96.70% (0.19%)
DoubletFinder	14,619	15%	-	78.71% (2.38%)	92.62% (0.30%)
DoubletFinder	14,619	15%	11.3%	66.81% (1.68%)	95.55% (0.20%)
DoubletFinder	14,619	21.9%	-	87.14% (1.69%)	85.96% (0.24%)
DoubletFinder	14,619	21.9%	16.6%	80.93% (2.15%)	91.55% (0.25%)

Tool	Cell Count	Rate	Normalization (theta)	Sensitivity (Std. Dev.)	Specificity (Std. Dev.)
Scrublet	6,525	10%	Z-score (man. 0.23)†	25.67% (0.59%)	95.66% (0.13%)
Scrublet	6,525	10%	Log (man. 0.30)	17.15% (0.38%)	97.82% (0.08%)
Scrublet	6,525	10.7%	Z-score (man. 0.25)†	25.63% (0.38%)	95.72% (0.11%)
Scrublet	6,525	10.7%	Log (man. 0.30)	18.20% (0.37%)	97.60% (0.11%)
Scrublet	6,525	12.5% (loading)†	Z-score (auto.)†	31.00% (2.32%)	93.94% (1.04%)
Scrublet	6,525	12.5% (loading)†	Log (man. 0.30)	21.24% (0.41%)	96.92% (0.06%)
Scrublet	6,525	15%	Z-score (auto.)†	27.61% (2.77%)	95.12% (0.84%)
Scrublet	6,525	15%	Log (man. 0.33)	23.11% (0.35%)	96.54% (0.07%)
Scrublet	6,525	21.9% (true)	Z-score (auto.)†	32.03% (1.52%)	93.54% (0.68%)
Scrublet	6,525	21.9% (true)	Log (man. 0.42)	24.05% (0.54%)	96.33% (0.07%)
Scrublet	14,619	10%	Z-score (auto)†	59.86% (1.11%)	94.68% (0.28%)
Scrublet	14,619	10%	Log (auto)	84.92% (0.29%)	92.51% (0.17%)
Scrublet	14,619	10.7% (true)	Z-score (auto)†	58.32% (0.92%)	95.12% (0.20%)
Scrublet	14,619	10.7% (true)	Log (auto)	85.57% (0.42%)	91.91% (0.26%)
Scrublet	14,619	12.5% (loading)†	Z-score (auto)†	59.61% (2.48%)	94.78% (0.74%)
Scrublet	14,619	12.5% (loading)†	Log (auto)	87.02% (0.48%)	90.61% (0.41%)
Scrublet	14,619	15%	Z-score (auto)†	62.38% (4.88%)	93.62% (2.00%)
Scrublet	14,619	15%	Log (auto)	87.29% (1.17%)	90.04% (1.27%)
Scrublet	14,619	21.9%	Z-score (man. 0.42)†	67.81% (0.39%)	91.49% (0.28%)
Scrublet	14,619	21.9%	Log (auto)	88.92% (0.76%)	87.56% (1.48%)

Table S1. **Relative performance of doublet removal tools, Related to Table 1.** Each indicated tool was applied ten times to the Demuxlet dataset with the indicated number of cells with different cell-gene expression filtering options. ρ': cluster merging parameter for reference dataset, high rank indicating option with the least cluster merging; minimum unique: number of uniquely expressed genes in a doublet cluster necessary for cluster to be "Rescued"; rate: putative doublet rate supplied to tool; homotypic adj: adjustment applied to reduce rate to account for (undetectable) homotypic doublets; normalization: count data either z-score or log transformed; theta: doublet score threshold for classifying a cell as a doublet, user-specified value or auto(matic) determination by tool. †=default value specified in original manuscript or online documentation (when applicable). See STAR METHODS for additional details.

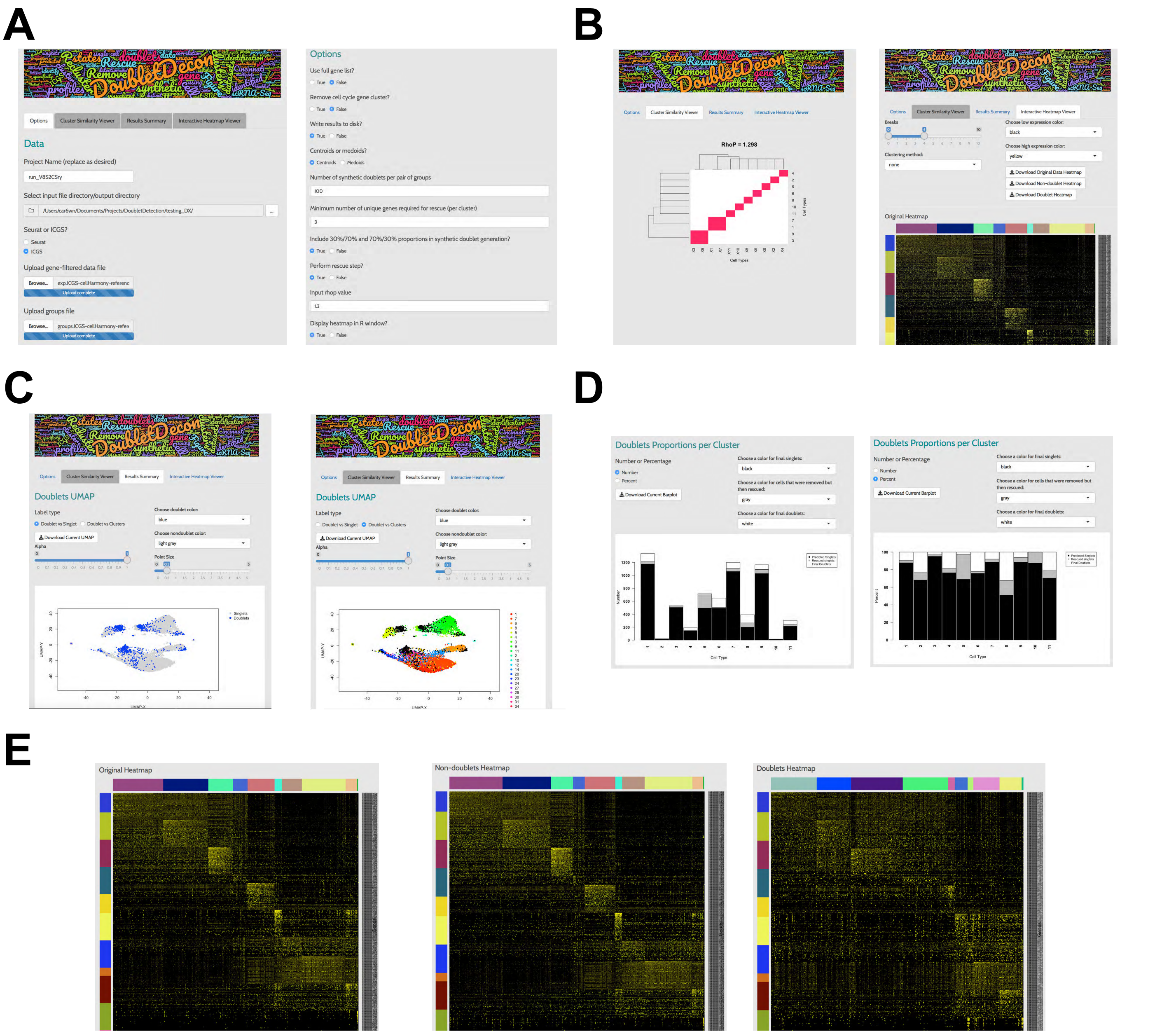

Figure S1. Interactive analysis, visualization and parameter tuning in the DoubletDecon graphical user interface, Related to the STAR Methods. Example usage of DoubletDecon is shown via the Shiny app for a PBMC dataset with cells from 8 separate donors with doublets detected by Demuxlet (Kang et al., 2018). A) Loading of input files from either ICGS or Seurat (left) and doublet detection parameters (parameters). The indicated tabs shown in the interface can be selected to adjust parameters or interactively view the data. B) Visualization of cluster similarity (centroid) to select the proper cluster merging threshold (ρ' values) to combine similar clusters prior to synthetic doublet creation (left). Here, a ρ' value 1.2-1.3 results in equivalent merging of clusters. The gene expression of markers for the original clusters are shown (right), with clusters re-labelled from x1-x11 (11 clusters). C) Visualization of the final predicted doublets (following "Rescue"), in a UMAP plot without (left) or with (right) indicated ICGS clusters. Predicted doublets typically will exist at the boundaries between clusters or at the cluster peripheries. D) Stacked bar charts of the number (left) and percentage (right) of cells in each cell cluster predicted to be doublets at the end of the end of the program, those that were initially predicted to be doublets but then rescued and singlets. E) Displayed interactive heatmap for the input scRNA-Seq cluster results prior to doublet removal (left) and after doublet removal for predicted singlets (middle) and predicted doublets (right).

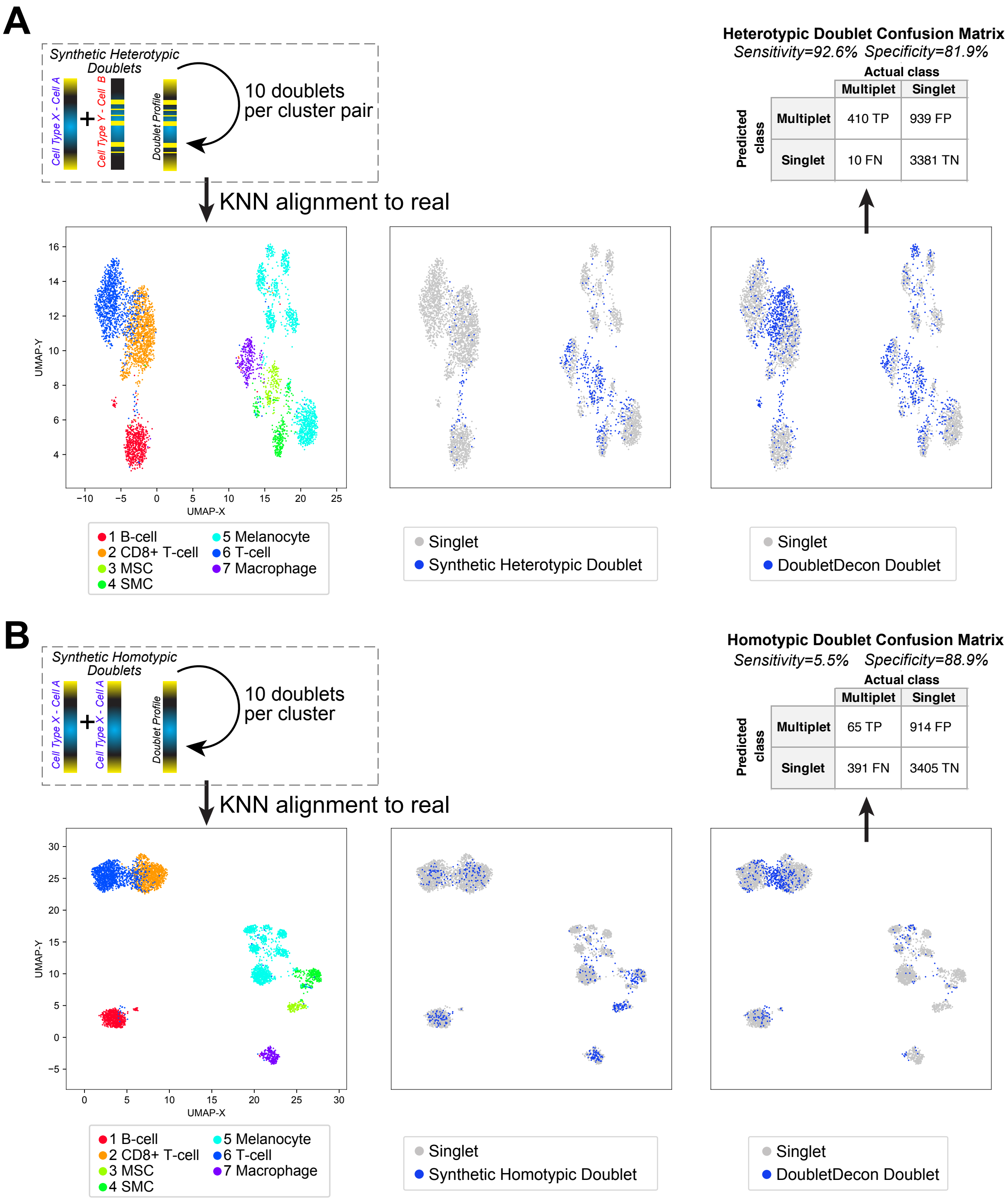

Figure S2. Detection of synthetic heterotypic doublets in human melanoma scRNA-Seq, Related to the STAR Methods. The analysis schema is shown for the evaluation of DoubletDecon on synthetic (A) heterotypic or (B) homotypic doublets of varying complexity (created with a 50/50 mix only). The same workflow used in DoubletDecon to produce synthetic doublets for doublet determination was applied in the creation of doublets for testing. Ten separately generated sets of synthetic doublets resulted in a sensitivity and specificity estimates (representative example shown in the displayed confusion matrix). UMAP plots derived from the ICGS clustering results shown for all annotated cell clusters (left), in silico derived synthetic doublets (middle) and DoubletDecon predicted doublets via deconvolution analysis (right). Labels for each cell population were independently derived through ICGS using its default cellular biomarker gene database via GO-Elite gene-set enrichment analysis.

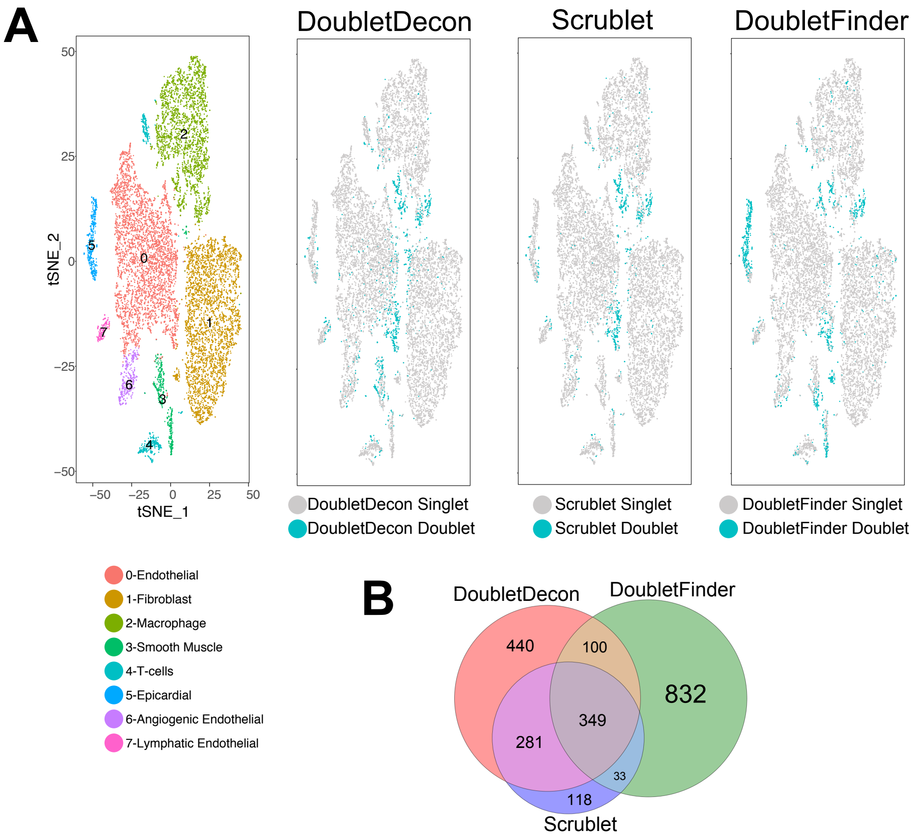

Figure S3. Common doublet cell population predictions in overloaded scRNA-Seq from mouse heart failure, Related to Figure 5. A) t-SNE visualization of the predominant cell populations identified by Seurat of ~13,000 heart cells collected via Drop-Seq. (Bottom) Labels for each cell population were independently derived through ICGS using its built-in cellular biomarker database gene-set enrichment analysis (GO-Elite function). Projected predicted doublets are shown for the three evaluated algorithms on projected into the t-SNE plot. B) Overlaps in doublet predictions for the three methods, indicating distinct overlapping subsets by all three algorithms. The parameters for Scrublet and DoubletDecon were according to the authors' recommendations or optimized for stability across runs.
