# Supplementary material for: DoubletDecon: Deconvoluting Doublets from Single-Cell RNA-Sequencing Data
Source: Cell Rep. Author manuscript; Available in PMC 2020 Jan 26. (PMC6983270; doi:10.1016/j.celrep.2019.09.082)
Supplement: 2 [file NIHMS1542667-supplement-2.pdf]

Cell Reports

Resource

DoubletDecon: Deconvoluting Doublets from Single-Cell RNA-Sequencing Data

Graphical Abstract

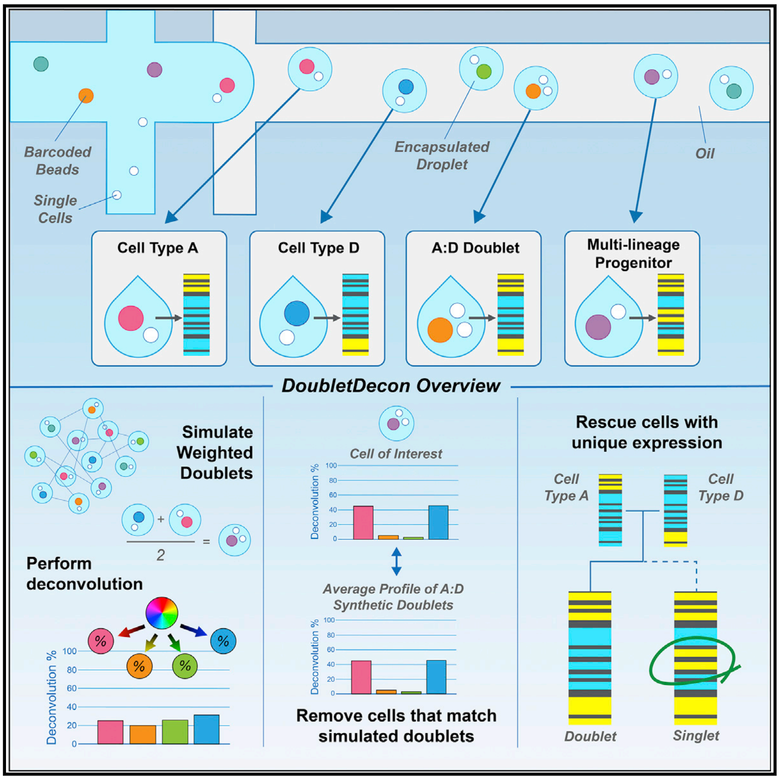

Highlights

- DoubletDecon uses deconvolution to identify and remove doublets in scRNA-seq data
- Retention of doublets can confound data analysis and cell population identification
- DoubletDecon limits erroneous removal of transitional and progenitor cells
- The algorithm identifies unique doublets relative to alternative approaches

Authors

Erica A.K. DePasquale, Daniel J. Schnell, Pieter-Jan Van Camp, ..., H. Leighton Grimes, Harinder Singh, Nathan Salomonis

Correspondence

nathan.salomonis@cchmc.org

In Brief

Multiplets are a source of confounding gene expression in single-cell RNA sequencing (scRNA-seq) that result from the simultaneous capture of multiple cells in a droplet. DePasquale et al. introduce DoubletDecon to identify putative doublets and to consider unique gene expression inherent to transitional states and progenitors to “rescue” singlet captures from inaccurate classification.

DePasquale et al., 2019, Cell Reports *29*, 1718–1727
November 5, 2019 © 2019 The Authors.
https://doi.org/10.1016/j.celrep.2019.09.082

CellPress

OPEN ACCESS
CellPress

Cell Reports
Resource

DoubletDecon: Deconvoluting Doublets from Single-Cell RNA-Sequencing Data

Erica A.K. DePasquale,[1,2] Daniel J. Schnell,[1,3] Pieter-Jan Van Camp,[1,2] Íñigo Valiente-Alandí,[3] Burns C. Blaxall,[3,4] H. Leighton Grimes,[4,5,6] Harinder Singh,[7,8,9] and Nathan Salomonis[1,2,4,10,*]
[1]Division of Biomedical Informatics, Cincinnati Children's Hospital Medical Center, Cincinnati, OH 45229, USA
[2]Department of Biomedical Informatics, University of Cincinnati, Cincinnati, OH 45221, USA
[3]Heart Institute and Center for Translational Fibrosis Research, Cincinnati Children's Hospital Medical Center, Cincinnati, OH 45229, USA
[4]Department of Pediatrics, University of Cincinnati, Cincinnati, OH 45221, USA
[5]Division of Immunobiology and Center for Systems Immunology, Cincinnati Children's Hospital Medical Center, Cincinnati, OH 45229, USA
[6]Division of Experimental Hematology and Cancer Biology, Cincinnati Children's Hospital Medical Center, Cincinnati, OH 45229, USA
[7]Center for Systems Immunology, University of Pittsburgh, Pittsburgh, PA 15260, USA
[8]Department of Immunology, University of Pittsburgh, Pittsburgh, PA 15260, USA
[9]Department of Computational and Systems Biology, University of Pittsburgh, Pittsburgh, PA 15620, USA
[10]Lead Contact
*Correspondence: nathan.salomonis@cchmc.org
https://doi.org/10.1016/j.celrep.2019.09.082

SUMMARY

Methods for single-cell RNA sequencing (scRNA-seq) have greatly advanced in recent years. While droplet- and well-based methods have increased the capture frequency of cells for scRNA-seq, these technologies readily produce technical artifacts, such as doublet cell captures. Doublets occurring between distinct cell types can appear as hybrid scRNA-seq profiles, but do not have distinct transcriptomes from individual cell states. We introduce DoubletDecon, an approach that detects doublets with a combination of deconvolution analyses and the identification of unique cell-state gene expression. We demonstrate the ability of DoubletDecon to identify synthetic, mixed-species, genetic, and cell-hashing cell doublets from scRNA-seq datasets of varying cellular complexity with a high sensitivity relative to alternative approaches. Importantly, this algorithm prevents the prediction of valid mixed-lineage and transitional cell states as doublets by considering their unique gene expression. DoubletDecon has an easy-to-use graphical user interface and is compatible with diverse species and unsupervised population detection algorithms.

INTRODUCTION

Single-cell genomics provides a powerful means to derive and ultimately characterize novel cell populations and transitional states (Olsson et al., 2016; Velten et al., 2017; Villani et al., 2017; Yanez et al., 2017). While single-cell profiling technologies continue to evolve at an astonishing pace, many challenges remain, including the separation of biological signal from technical noise. A common source of confounding gene expression in single-cell RNA-sequencing (scRNA-seq) is the occurrence of multiplet cell profiles that result from the simultaneous capture of multiple cells in a single well or droplet (Kang et al., 2018).

As demonstrated by species mixing experiments, the frequency of mutliplets increases with greater loading of cells for droplet-based scRNA-seq platforms (Goldstein et al., 2017; Gong and Szustakowski, 2013; Macosko et al., 2015; Stoeckius et al., 2018). As a result, researchers are often advised to load fewer cells to decrease the occurrence of multiplets, hence limiting the cellular depth afforded by these technologies. Beyond the simultaneous capture of multiple cells due to a high concentration of cells, insufficient dissociation will increase the frequency of aggregates and subsequently multiplet captures. Doublets, multiplets with two captured cells, can be grouped into two main classes: (1) those that occur between transcriptionally distinct cell types (heterotypic) and (2) those that occur within the same cell type (homotypic), with multiplets of more than two cells being exceedingly rare (0.36%, assuming a doublet rate of 8%). Experimental methods, such as Cell Hashing, aim to address the challenge of doublet identification by labeling cells with different oligonucleotide bar codes to remove artifacts, but they are costly, increase the likelihood of cell death, and cannot be applied to previously generated datasets (Stoeckius et al., 2018).

Retention of doublets can significantly confound the analysis and interpretation of scRNA-seq data, in particular the identification of novel cell states, developmental trajectories, and mixed-lineage progenitors (Magella et al., 2018; Olsson et al., 2016). Mixed-lineage cells include multi-lineage progenitors, which coincidently prime markers for multiple lineages and that exist at bifurcations within *in silico* developmental trajectories (Chen et al., 2019; Lu et al., 2018; Olsson et al., 2016). As such, the spatial location and shared gene expression of these cells with others complicate doublet detection methods that rely solely on their similarity to synthetic doublets for identification. Hence, the erroneous exclusion of such mixed-lineage populations can hinder the unbiased evaluation of progenitor hierarchies in healthy cells and disease states. Conversely, the inappropriate

 © 2019 The Authors.
This is an open access article under the CC BY license (http://creativecommons.org/licenses/by/4.0/).

retention of doublets can confound single-cell analyses in which refined clustering is used to establish novel cell states (i.e., doublet cell clusters).

While the need for specialized *in silico* doublet removal methods is evident, there remain many biological and computational challenges. First, multiplet detection is confounded by varying degrees of sparsity of the transcriptomic data, with as little as a few hundred unique molecular identifiers (UMIs) for a single-cell transcriptome, resulting in poor correlation to comparable bulk RNA-seq profiles (Kashima et al., 2018; Mantsoki et al., 2016). Although multiplets should have a distinct global distribution of genes and UMI counts, with twice the RNA content, these variables are insufficient to accurately predict which cells are doublets on their own (Stoeckius et al., 2018). Furthermore, differing RNA abundance and/or technical variation in cDNA generation may result in uneven contribution from each cell. Hence, modeling doublets as an equal contribution of two different cells is likely to be overly simplistic. Two recently developed methods, DoubletFinder and Scrublet, approach the problem from a synthetic doublet nearest-neighbor strategy to find hybrid transcriptomes (McGinnis et al., 2019; Wolock et al., 2019). While these methods have high reported accuracy, the authors note that algorithm performance is highly dependent on the selection of appropriate parameters, such as the expected doublet rate, which is not always known. Additionally, these methods do not explicitly consider the added complication of transitional and mixed-lineage cell states, which can possess hybrid transcriptomes.

Here, we describe a deconvolution-based strategy to remove heterotypic doublets while preserving transitional and progenitor cell states. Our approach, DoubletDecon, applies nonnegative decomposition, a deconvolution method originally designed to estimate cell-type proportions in bulk RNA-seq data, to single-cell datasets to assess the underlying contribution of concurrent gene expression programs within a single-cell library. This approach compares the proportional makeup of each cell, termed here as the deconvolution cell profile (DCP), to all cell clusters in the dataset to find those that match one of many possible synthetic doublet combinations. DoubletDecon employs marker genes and cell clusters from well-established unsupervised scRNA-seq workflows, including Iterative Clustering and Guide-gene Selection (ICGS) and Seurat, as reference states for deconvolution (Olsson et al., 2016; Satija et al., 2015). To overcome the specific computational challenges associated with the detection of doublets, DoubletDecon includes three approaches not present in alternative tools. To account for unequal contribution of the originating cell transcriptomes during doublet formation, synthetic doublets are generated by either an average of two cells from distinct clusters in the dataset or with an additional set of weighted synthetics with 30%/70% contribution from the cells. DoubletDecon also accounts for the presence of transcriptionally similar clusters, an often unintended result of unsupervised clustering methods, by cluster merging to define discrete cell types for use as deconvolution references. Finally, to improve the accuracy of its predictions, DoubletDecon considers unique gene expression inherent to biologically valid transitional states and progenitors to "rescue" singlet captures from inaccurate classification as doublets.

We demonstrate the power of this approach to identify real, synthetic, and biologically confounding doublet cells in diverse scRNA-seq datasets of varying size and complexity. We further provide guidelines to users for best-practice application of this this software and discuss its applicability to diverse scRNA-seq datasets. Finally, we performed comprehensive benchmarking of multiple doublet detection algorithms to provide guidance on the choice of appropriate tools and parameters for doublet removal.

RESULTS

Overview

To detect heterotypic doublet captures and distinguish them from gradual cellular transitions, we developed a multi-step analysis strategy that identifies an initial set of putative doublets based on deconvolution analysis, then rescues erroneously predicted doublet clusters that have unique gene expression (STAR Methods; Figure 1A). The program first calculates centroids based on previously defined cell clusters from supervised or unsupervised methods to create distinct deconvolution references. During the creation of references for deconvolution, DoubletDecon accounts for the presence of transcriptionally similar cell clusters through cluster merging (Figure 1B). Next, DoubletDecon creates a deconvolution cell profile, or DCP, containing the percentage estimates of the contribution of each reference cell state, each of which sums to 100% (Figure 1C). In the initial "remove" step, cells whose DCP is most similar to the DCP of generated synthetic cell clusters are considered putative doublets. These cells are removed from their original clusters and regrouped by their top deconvolution contributors in the "recluster" step. Finally, putative doublet clusters that have gene expression patterns not prevalent in the original clusters are returned to their initial clusters, with the remaining cells being labeled as doublets in the "rescue" step. To evaluate the performace of DoubletDecon in diverse use cases, we selected test datasets with distinct biological and technical challenges (Figure 1D). We used input data from both ICGS and Seurat workflows, in which unique cell states and cell-state-associated genes sets were already defined or re-derived, with a variety of cell filtering options and parameters. In the evaluation of these datasets we optimized selection of the cluster merging statistic (ρ') to visually merge similar clusters in the DoubletDecon graphical interface prior to optimizing synthetic doublet creation. A detailed description of the DoubletDecon algorithm is included in the STAR Methods, along with information on datasets, evaluations, parameter tuning, possible limitations of the approach, and instructions for using the graphical user interface (Figure S1).

Identification of Doublets from Species Mixing Experiments

As a first evaluation of DoubletDecon's ability to identify doublets, we considered a mixed-species scRNA-seq experiment. Analysis of a publicly available dataset of murine NIH 3T3 and human HEK293 cells, mixed at roughly equal proportions and profiled by 10x Genomics produced ~6% heterotypic doublets that are clearly separable from their single-species counterparts by principal-component analysis (PCA) (Figure 2A). Output of the

OPEN
ACCESS
CellPress

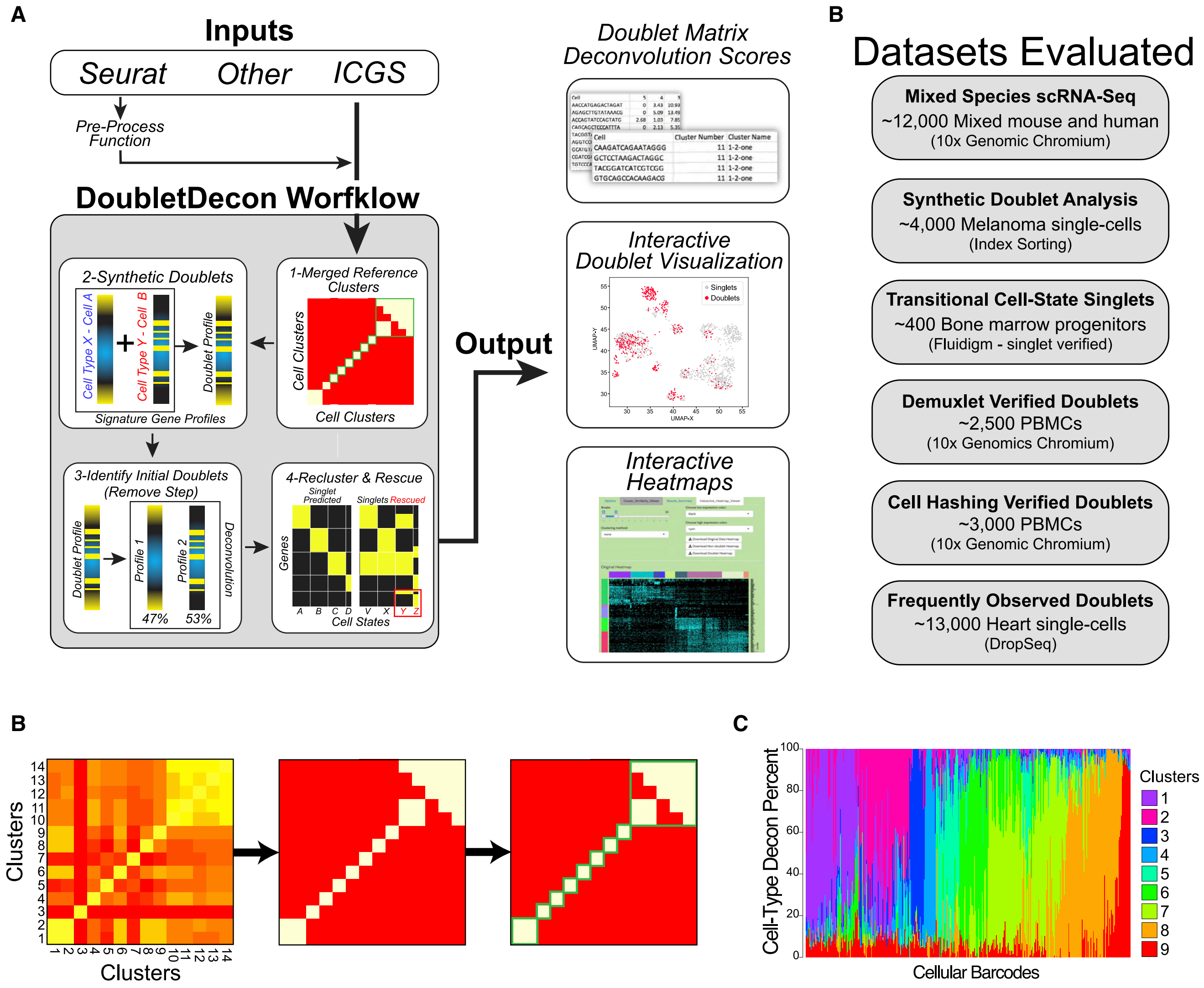

Figure 1. Deconvolution and Detection of Cell Doublets with DoubletDecon

(A) Outline of the broad steps employed by DoubletDecon, including cluster merging, synthetic doublet generation, deconvolution, and rescue of initially predicted doublets through unique gene expression identification. The principal file inputs and sources are indicated along with distinct tabular and graphical outputs from the DoubletDecon package in R or through an easy-to-use graphical interface.

(B) Illustration of cluster similarity determination from DoubletDecon to determine the threshold for cluster merging prior to synthetic doublet creation and deconvolution. Each centroid is calculated from the average gene expression of each separate cell state for all algorithm-selected cell-state marker genes (e.g., Seurat, ICGS). Initially, a centroid or medoid correlation matrix is created (left). Next, a threshold for centroid or medoid similarity is defined by the formula for ρ (outlined in the STAR Methods), with the user-defined value of ρ' used to set the level of similarity required for a cluster to be considered correlated (middle). Finally, this new binary correlation matrix is visualized with a heatmap and Markov clustering is used to determine which sets of clusters should be merged for multiplet detection (right).

(C) The frequency of cell-state deconvolution profiles is shown for a dataset without doublets (microscopy validated) (Olsson et al., 2016). Each column represents a different cell, in which each color indicates the percentage contribution of a reference cell type for that cell. Note, the majority are predicted to be composed principally of a single-cell-type reference.

(D) Datasets evaluated to assess DoubletDecon's accuracy on gene expression evidenced doublets with the number of cells and method of single-cell capture.

deconvolution step in DoubletDecon can be summarized as a value between 0% and 100% for each original cluster, in this case mouse and human, for each cell. Visualization of the deconvolution results within the original PCA shows an expected selective enrichment for each of the single-species clusters and overlapping intermediate results within mouse-human hybrid cells (Figure 2B). When only the mouse-human hybrids are considered, we observe a bimodal distribution of deconvolution with peaks at 30% human and 70% mouse, instead of the expected 50% split (Figure 2C). As anticipated, the deconvolution results for single-species mouse or human cells were heavily skewed toward 100% (Figure 2D). As expected from the

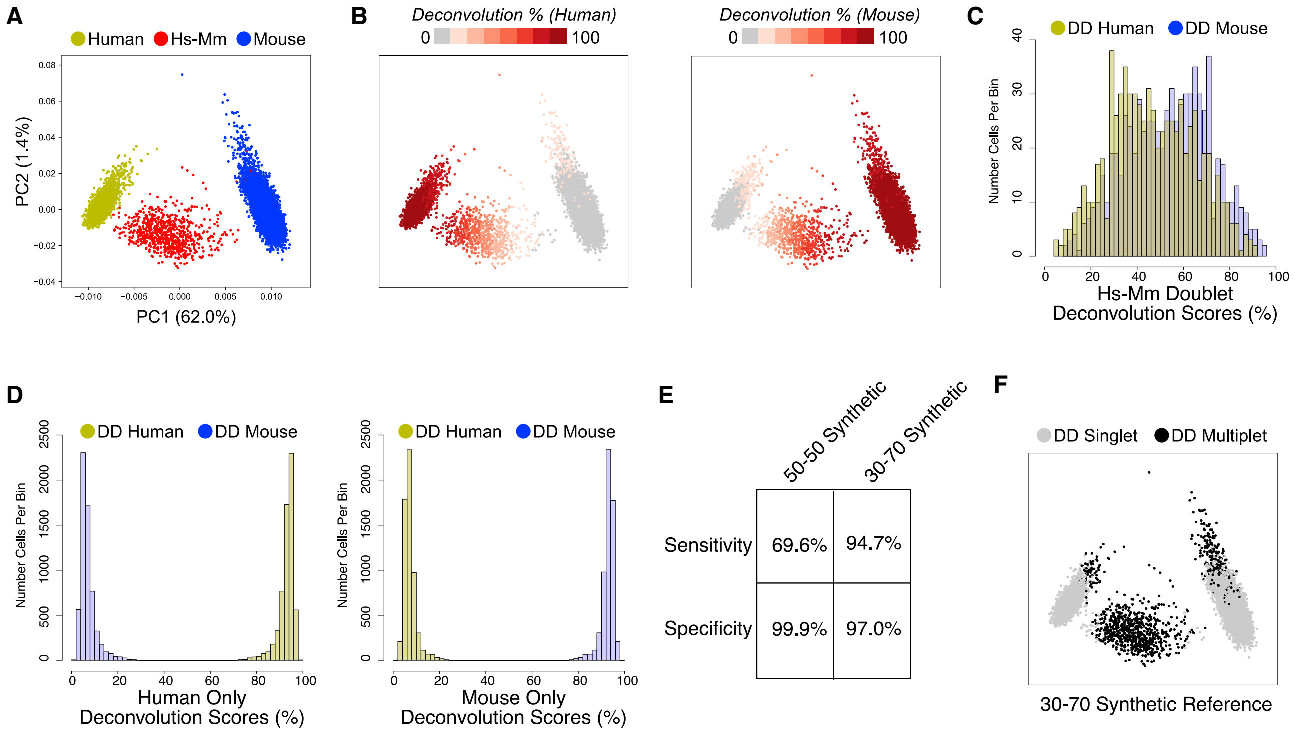

Figure 2. DoubletDecon Readily Distinguishes Experimentally Validated Doublets in Species-Mixing scRNA-Seq

(A) Separation of mouse, human, and mixed-species doublet scRNA-seq profiles by principal-component analysis (PCA) of ICGS variable genes. Species assignments are defined by the total number of aligned reads to either human (yellow), mouse (blue), or both (red) genomes.

(B) Projection of species-specific deconvolution results (against human or mouse ICGS clusters) are displayed along the same PCA plot. Cells in gray indicate <10% identify to the indicated cluster, >90% in dark red, and lighter shades of red indicating intermediate scores.

(C) Histogram of the mouse (blue) and human (yellow) DCP results (x axis) for known species mixed cells, indicating a bi-modal distribution for deconvolution scores peaking at 30% and 70%.

(D) The same histogram is shown for deconvolution scores in only human cells (left) and only mouse cells (right), indicating a skewed distribution toward the correct species.

(E) The accuracy of DoubletDecon doublet predictions using synthetic reference doublets derived from either a 50/50 equal contribution of cell transcriptomes ("only50" parameter) or from weighted averages of 30/70 and 70/30, in addition to the 50/50 synthetic doublets.

(F) Projection of final called doublets (black) in the PCA, using 30/70 synthetic doublets.

distributions of these DCPs, using synthetic doublets with a 30/70 weighted average detects mixed-species cells with a relatively high accuracy (~95% sensitivity and ~97% specificity), whereas use of 50/50 synthetic doublets alone results in close to 100% specificity at the cost of reduced doublet sensitivity (~70%) (Figures 2E and 2F). We note that cells that were incorrectly classified as mouse-human doublets in the 30/70 analysis have ~30% fewer expressed genes (normalized counts/gene > 1, t test $p < 0.001$) than other single-species cells, indicating that poorly sequenced singlets have an increased likelihood to be called doublets.

Evaluation of Synthetic Doublets from Complex Tissue

While DoubletDecon accurately identified doublets in a mixed-species dataset, this example is not representative of typical scRNA-seq data, which frequently has subtle cell-state differences and more than two populations. To assess the ability of DoubletDecon to detect homotypic and heterotypic doublets in a more realistic example, we produced a synthetic doublet evaluation dataset in which both heterotypic and homotypic doublets could be generated and separately assessed. This dataset comprised four human immune and three melanocyte tumor populations (SMART-Seq2 protocol, index sorting) (Tirosh et al., 2016), with synthetic doublets integrated into existing cell clusters using the recently developed label projection approach, cellHarmony (STAR Methods, Evaluation Dataset Processing Parameters). Consistent with the mixed species results, heterotypic synthetic doublets were identified with high sensitivity (average 90.7% ± 1.2%) and moderate-to-high specificity (average 82.7% ± 0.9%) (Figure S2A), while synthetic homotypic doublets could not be effectively detected (average sensitivity of 5.6% ± 0.2%) (Figure S2B). Though homotypic doublets are not detected using DoubletDecon or alternative methods, the presence of these artifacts does not appear to impede the identification of valid cell populations, making homotypic doublets less problematic than heterotypic doublets. Importantly, false positives in this analysis will be overestimated, contributing to the relatively lower specificity, as real doublets exist in this dataset but are not annotated as doublets by the original dataset authors.

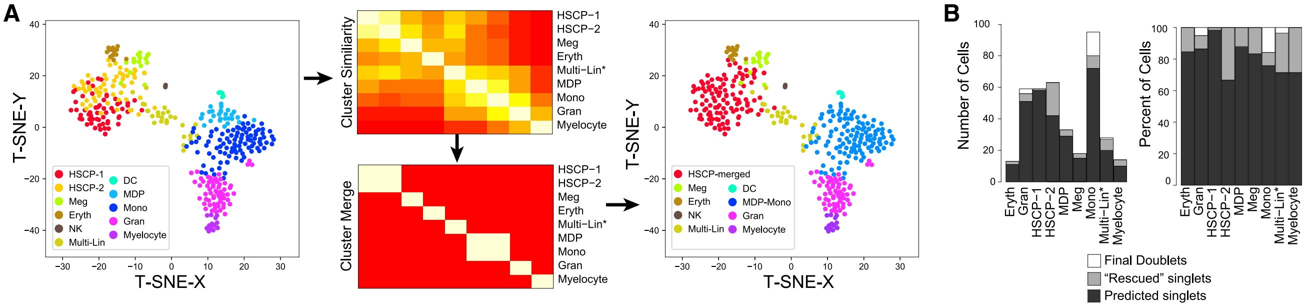

Figure 3. Recovery of Rare Transitional Cell States through Singlet Rescue

Evaluation of a scRNA-seq dataset of mouse hematopoietic progenitors, with rare transitional states, is shown. All initially detected multiplets were removed through a microscopy validation step to selectively evaluate specificity for doublet detection.

(A) Identification of highly related clusters for DoubletDecon reference creation from the original ICGS unsupervised population predictions (Olsson et al., 2016). (Left) Highlighted ICGS cell populations within a t-Distributed Stochastic Neighbor Embedding (t-SNE) before cluster merging. (Middle) DoubletDecon cluster similarity heatmaps indicating similarity and clustering merging. (Right) t-SNE plot of the merged cell populations.

(B) Bar graph displaying number of cells within each cluster that were never removed (dark gray, "predicted singlets"), removed during the "remove" step but were subsequently rescued (light gray, "rescued singlets"), and removed during the "remove" step and were not rescued (white, "final doublets) per total cells in each cluster (left) and percentage of cells in each cluster (right).

Rescue of Transitional Cell States Predicted as Doublets

As previously demonstrated, developmental and progenitor cell specification hierarchies inherently contain cells with transitioning gene expression and mixed-lineage cell states (Lu et al., 2018; Magella et al., 2018; Olsson et al., 2016). We postulate that such states may result in frequent false-positive doublet predictions when the unique gene expression intrinsic to those populations is not considered during doublet identification. Analysis of a published hematopoietic dataset of 383 cells over eight independent cell captures using the Fluidigm technology in parallel with microscopic doublet cell exclusion was used as validation of DoubletDecon's ability to retain these transitioning and mixed-lineage cells. Importantly, this dataset is enriched in experimentally validated transitional and multilineage cell populations, appearing as hybrids, representing a continuum of divergent differentiation cell states in mouse bone marrow. Given the high similarity of clusters called by ICGS, we selected a cluster merging threshold (ρ') that led to merging of the HSCP-1 and HSCP-2 (HSCP-merged) and the monocyte and macrophage-dendritic cell precursor (MDP-Mono) clusters (Figure 3A; STAR Methods). Using a 50/50 average for synthetic doublet generation, as recommended for datasets with a small percentage of expected doublets, this evaluation results in 80.7% specificity in the initial doublet detection step, which increases to 95.0% when unique gene expression is considered (Figure 3B). These data suggest that rescue of erroneously predicted doublets is necessary to retain transitional cell states.

Identification of Experimentally Verified Doublets from Mixed-Donor PBMCs

As further validation of DoubletDecon, we analyzed two recently described human peripheral blood mononuclear cell (PBMC) datasets in which multiplets were experimentally defined using either (1) donor SNP information with the software Demuxlet (Kang et al., 2018) or (2) through selective antibody-mediated oligonucleotide labeling via Cell Hashing (Stoeckius et al., 2018). In these experiments, the research teams intentionally overloaded a single 10x Chromium port with cells from eight independent donors to yield a high proportion of doublet cell captures (> 10%). For both datasets, we only considered cellular bar codes with a minimum number of genes and UMIs expressed consistent with well-accepted guidelines in the field (STAR Methods, Evaluation Dataset Processing Parameters). When evaluated by DoubletDecon, analysis of the Demuxlet-annotated dataset identified verified doublets with a mean (SD) sensitivity of 56.9% (2.1%) and a specificity of 81.2% (1.2%) over 10 independent trials. Specificity in the Cell Hashing dataset was similar to that in Demuxlet, 82.4% (0.6%), while sensitivity averaged 38.1% (1.2%) (Figures 4A and 4B; Table 1). Though the recall in these datasets is relatively low, this result is expected, as homotypic doublets are prevalent in this dataset and experimentally indistinguishable from heterotypic doublets. Comparison of our doublet predictions to those from two other doublet detection tools, Scrublet and DoubletFinder, reveals that DoubletDecon uniquely identifies hundreds of true-positive doublets (Figures 4C and 4D), with improved sensitivity and slightly decreased specificity when using default parameters and suggested filtering (Table 1). As the authors of Scrublet, and DoubletFinder have reported different doublet exclusion performance on this Demuxlet dataset, we reanalyzed this dataset with a range of software parameters and dataset filtering options. While the performance of DoubletDecon remained largely stable across the evaluated parameters (ρ' and min_uniq), DoubletFinder and Scrublet varied widely in reponse to different UMI filtering cutoffs, expected doublet rate, and non-default programmatic options (Table S1). While varying these options significantly improves sensitivity for Scrublet and DoubletFinder, without *a priori* knowledge of known doublets for parameter tuning, obtaining such results would remain extremely problematic for the end user.

An alternative to running independent doublet detection methods is a consensus approach, in which the results from

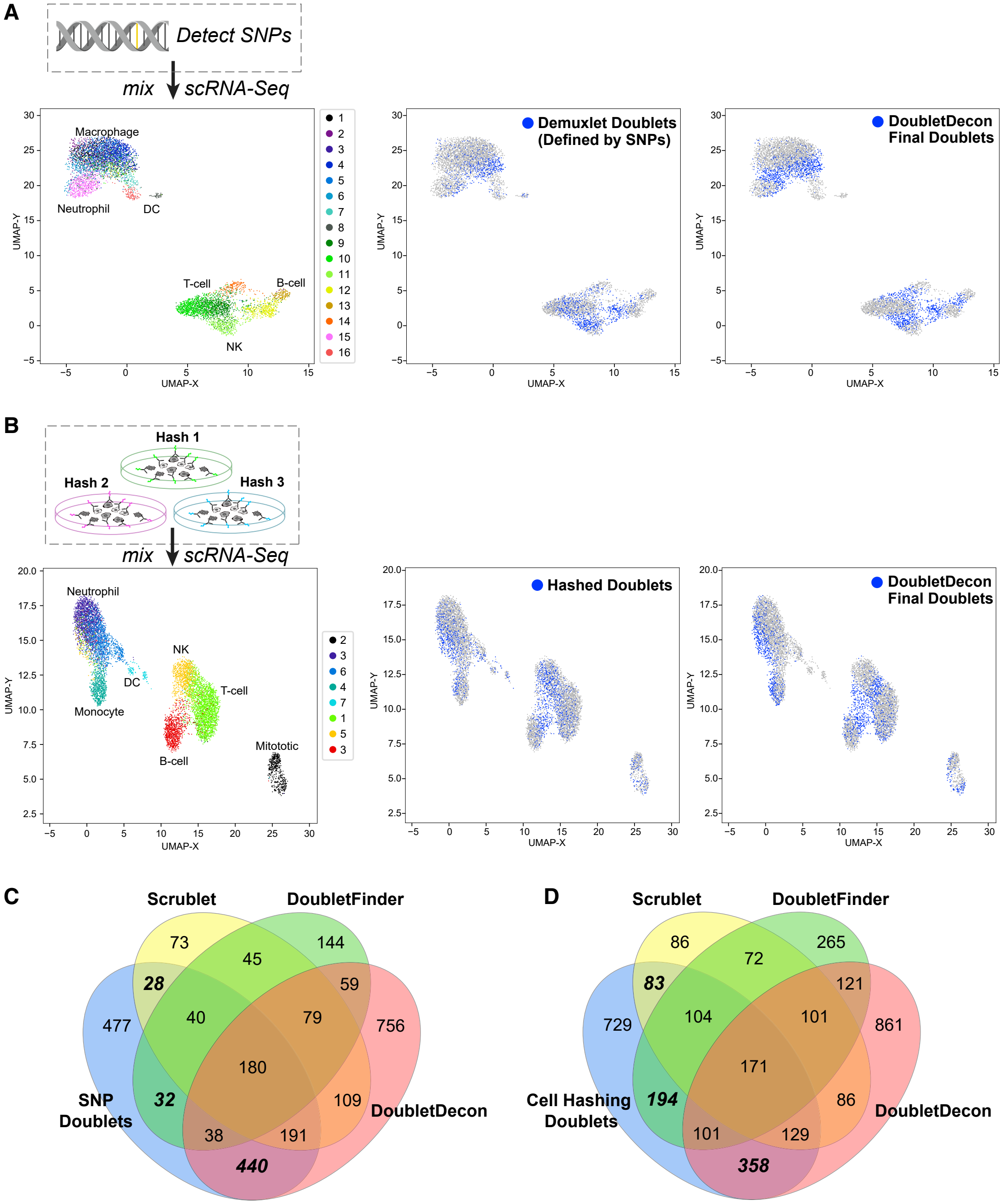

Figure 4. Detection of Experimentally Validated Doublets from Peripheral Blood Mononuclear Cells (PBMCs)

(A and B) The analysis schema is shown for the evaluation of DoubletDecon on *in silico* identified doublet cell profiles obtained from the (A) Dexmulet software and (B) the Cell Hashing protocol. Demuxlet identifies cells with a combination of genomic variants associated with the eight profiled single-cell donors to find cellular bar codes with hybrid genotype profiles, whereas Cell Hashing selectively labels all cells from a single sample (donor) using different oligonucleotides conjugated to a common antibody. (Left) A Uniform Manifold Approximation and Projection (UMAP) plot of the *de novo* clusters obtained from analysis with ICGS. (Middle) UMAP projection of Demuxlet called doublets are indicated in blue. (Right) UMAP projections of DoubletDecon-classified doublets are highlighted in blue. Labels for each cell population were independently derived through ICGS version 2.0 using a published database of hematopeotic and immune markers via GO-Elite gene set enrichment analysis (Hay et al., 2018).

(C and D) Venn diagrams representing the number of overlapping doublet predictions from the software packages DoubletDecon, Scrublet, and DoubletFinder on two previously published datasets of overloaded donor PBMCs using the (C) Demuxlet or (D) Cell Hashing protocols using the same filtered datasets described above. Hashing doublets, doublets defined from distinct hashtag oligo (HTO). If two or more HTOs had >20% of the total hashtag reads, they were considered multiplets (4,200 out of the initial total 12,000 cellular bar codes). Demuxlet doublets, doublets identified by Kang et al. (2018) using the software Demuxlet.

Table 1. Relative Performance of Doublet Removal Tools

Dataset	Tool	Parameters	Sensitivity, % (SD, %)	Specificity, % (SD, %)
Demuxlet n = 6,525	DoubletDecon	rhop = 1.2, min_uniq = 4	56.90 (2.1)	81.20 (1.2)
Demuxlet n = 6,525	DoubletFinder	10 PCs, pN = 0.25, pK = auto, rate = 12.5% - homotypic adj.	21.31 (2.8)	93.87 (0.8)
Demuxlet n = 6,525	Scrublet	25 PCs, top 25% HVGs, rate = 12.5%, *Z* scores, theta = auto	31.00 (2.3)	93.94 (1.0)
Cell Hashing n = 8,402	DoubletDecon	rhop = 1.2, min_uniq = 4	38.10 (1.2)	82.40 (0.6)
Cell Hashing n = 8,402	DoubletFinder	10 PCs, pN = 0.25, pK = auto, rate = 20% - homotypic adj.	32.23 (3.8)	89.42 (1.1)
Cell Hashing n = 8,402	Scrublet	30 PCs, top 15% HVGs, rate = 20%, *Z* scores, theta = 0.37	26.16 (0.6)	94.70 (0.2)

Each indicated tool was applied 10 times to the Demuxlet or Cell Hashing dataset with the indicated number of cells (filtered for number of UMIs and genes). rhop, cluster merging parameter for reference dataset; min_uniq, number of uniquely expressed genes in a doublet cluster necessary for cluster to be "rescued"; PCs, principal components; pN, controls number of simulated doublets added to dataset; pK, controls size of neighborhood used to compute doublet score, user-specified value, or auto(matic) determination by tool; rate, putative doublet rate supplied to tool; homotypic adj, adjustment applied to reduce rate to account for (undetectable) homotypic doublets; HVGs, highly variable genes; *Z* scores, count data centered and scaled; and theta, doublet score threshold for classifying a cell as a doublet, user-specified value, or auto(matic) determination by tool. See STAR Methods for additional details and Table S1 for additional performance results for the same datasets with different cell-gene expression filtering options.

multiple algorithms are compared or a single method is rerun multiple times. Such approaches can in principle be used to favor higher sensitivity or higher specificity, depending on the specific goals of the analysis. To test this assertion, we use the F1 score as a measure of overall performance given the uneven class distribution in these evaluation datasets. We find that the union of all three doublet detection algorithms (DoubletDecon, DoubletFinder, and Scrublet) increases sensitivity and gives the highest F1 score (0.52 and 0.50) when compared with any two methods combined or individual methods alone (Table 2). Specificity can be further improved by intersecting doublet calls, which results in lower sensitivity than each method applied on its own. Alternatively, specificity can be increased by performing multiple runs of DoubletDecon with the same parameters. Because of the random nature of synthetic doublet generation, not every run of DoubletDecon will produce exactly the same doublet calls. By running the algorithm 20 times and selecting only those cells that are predicted as doublets all 20 times, specificity nears 90% at a moderate loss of sensitivity (Table 2). This provides another option for users who wish to prioritize higher specificity.

Resolving Disease-Associated Cell States

An important application of doublet removal is the identification of biologically valid cellular heterogeneity among transcriptionally related cell types. One such example is the identification of discrete cell states within healthy or diseased tissue. The presence of heterotypic doublet cells directly impedes this process, as unsupervised analysis tools cannot easily distinguish between valid cellular heterogeneity and contamination. To this end, we performed overloaded droplet-based single-cell profiling on ~13,000 heart cells from a surgical model of heart failure (transverse aortic constriction). Our initial analysis with the software Seurat identified eight transcriptionally distinct clusters corresponding to well-defined heart populations, with the exception of cardiomyocytes not effectively captured by droplet-based methods due to size (Figure 5A). DoubletDecon predicted 1,170 doublets, localized to the peripheries of the major cell clusters. Notably, similar doublet-enriched populations were observed with the tools Scrublet and DoubletFinder in this dataset, though DoubletFinder erroneously predicted epicardial cells as a doublet cluster (Figure S3A). To explore the specific impact of doublets on the identification of cell states within a well-defined cell type, we focused on the largest cluster of cells (endothelial, n = 4,411), with 375 DoubletDecon-predicted doublets. Prior to removal of these predicted doublets, a distinct doublet cluster was identified, marked by high *Postn* gene expression, a specific marker of injury-associated profibrotic fibroblasts (Figure 5B). However, when Seurat was rerun on the endothelial cell cluster without predicted doublets, this same doublet population was not observed, as evidenced by no consistent *Postn* expression among the identified clusters, which resulted in more accurate cell-state predictions (Figure 5C). These data empirically demonstrate the importance of accurate heterotypic doublet exclusion on identifying cell identity programs without prior knowledge. Finally, to assess the stability of DoubletDecon's predictions, we re-analyzed the same heart scRNA-seq dataset using lower and higher Seurat resolutions to generate fewer (5) and more (11) clusters with the same cluster merging threshold (ρ' = 1.5). Importantly, the majority of the detected doublets were retained across these different resolutions, indicating that such predictions are largely stable for different unsupervised clustering solutions (Figure 5D).

DISCUSSION

As the number and size of single-cell datasets increases, standardized multiplet discovery workflows are necessary to remove technical artifacts that can confound the identification of valid cell states. DoubletDecon takes advantage of existing unsupervised population detection approaches, such as Seurat and ICGS, to model doublet gene expression profiles. Our approach is applicable to both large and small datasets with both discrete

Table 2. Performance of Combinations of Doublet Removal Tools

Cell Hashing Doublets								
Tools	Combination	TPs	FPs	TNs	FNs	Sensitivity	Specificity	F1
DoubletDecon	–	759	1,169	5,364	1,110	40.61	82.11	0.40
DoubletFinder	–	570	559	5,974	1,299	30.5	91.44	0.38
Scrublet	–	487	345	6,188	1,382	26.06	94.72	0.36
DoubletDecon and DoubletFinder	union	1,057	1,506	5,027	812	56.55	76.95	0.48
DoubletFinder and Scrublet	union	782	731	5,802	1,087	41.84	88.81	0.46
DoubletDecon and Scrublet	union	946	1,327	5,206	923	50.62	79.69	0.46
All three methods	union	1,140	1,592	4,941	729	61	75.63	0.50
DoubletDecon and DoubletFinder	intersection	272	222	6,311	1,597	14.55	96.6	0.23
DoubletFinder and Scrublet	intersection	275	173	6,360	1,594	14.71	97.35	0.24
DoubletDecon and Scrublet	intersection	300	187	6,346	1,569	16.05	97.14	0.26
All three methods	intersection	171	101	6,432	1,698	9.15	98.45	0.16
DoubletDecon	20 runs	510	705	5,828	1,359	27.29	89.21	0.33
Demuxlet Doublets								
Tools	Combination	TP	FP	TN	FN	Sensitivity	Specificity	F1
DoubletDecon	–	849	1,003	4,096	577	59.54	80.33	0.52
DoubletFinder	–	290	327	4,772	1,136	20.34	93.59	0.28
Scrublet	–	439	306	4,793	987	30.79	94	0.40
DoubletDecon and DoubletFinder	union	921	1,192	3,907	505	64.59	76.62	0.52
DoubletFinder and Scrublet	union	509	509	4,590	917	35.69	90.02	0.42
DoubletDecon and Scrublet	union	917	1,121	3,978	509	64.31	78.02	0.53
All three methods	union	949	1,265	3,834	477	66.55	75.19	0.52
DoubletDecon and DoubletFinder	intersection	218	138	4,961	1,208	15.29	97.29	0.24
DoubletFinder and Scrublet	intersection	220	124	4,975	1,206	15.43	97.57	0.25
DoubletDecon and Scrublet	intersection	371	188	4,911	1,055	26.02	96.31	0.37
All three methods	intersection	180	79	5,020	1,246	12.62	98.45	0.21
DoubletDecon	20 runs	664	629	4,470	763	46.53	87.66	0.49

The number of overlapping doublet predictions from the software packages DoubletDecon, Scrublet, and DoubletFinder on two previously published datasets of overloaded donor PBMCs using the Demuxlet or Cell Hashing protocol. DoubletDecon was also tested with 20 test runs, with cells called as doublets all 20 times being considered DoubletDecon doublets for statistical analyses. Cell Hashing doublets, doublets defined from distinct hashtag oligo (HTO). If two or more HTOs had > 20% of the total hashtags reads, they were considered multiplets (4,200 out of the initial total 12,000 cellular bar codes). Demuxlet doublets, doublets identified by Kang et al. (2018) using the software Demuxlet. TPs, true positives (true doublets); FPs, false positives; TNs, true negatives (true singlets); FNs, false negatives; and F1, F1 score. See STAR Methods for additional details.

cell populations and gradual cellular transitions, by automatically grouping correlated cell states. This approach is methodologically distinct from alternative solutions by considering each cell as the decomposition of all possible reference cell populations. Given that valid hybrid transcriptomic states exist throughout development, such as transitional cell states and bi-potential intermediates, DoubletDecon includes specialized methods to rescue preliminarily removed cell clusters that include unique gene expression patterns. As demonstrated here, this method can significantly reduce the number of doublets that are known false positives.

Although DoubletDecon is able to effectively identify and exclude a high proportion of multiplet captures, we believe that this method can be further exploited to identify additional unwanted and desired sources of variation. False negatives with this approach currently include extremely rare multiplets of more than two cells, as well as doublets of highly similar cell states. We aim to enable the discovery of such multiplets in the future, which theoretically should be identifiable using our existing deconvolution-based strategy. These analyses demonstrate the importance of doublet exclusion in diverse biological use cases, from clarifying population heterogeneity to protection against removal of transitional cell states. As with other computational doublet exclusion methods, proper parameter tuning remains a critical determinant of performance. Here, we comprehensively benchmark multiple doublet detection algorithms, providing the user with guidance on the choice of tools and associated parameters. We further provide guidelines to users for best-practice application of this software and discuss its applicability to diverse scRNA-seq datasets and research questions (STAR Methods; Parameter Tuning and Potential Limitations). DoubletDecon's parameters can be easily tuned through an intuitive graphical user interface to iteratively evaluate the impact on putative doublet cells. As noted in these recommendations, while this tool performs well in diverse test datasets, DoubletDecon relies on a number of assumptions

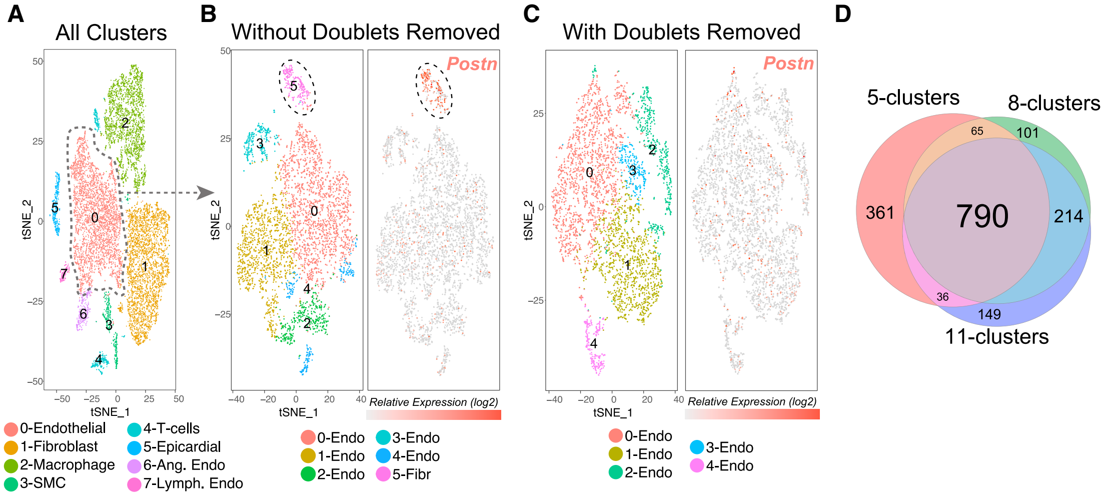

Figure 5. Empirical Removal of Confounding Doublet-Cell Populations for Unsupervised Subtype Detection

(A) t-SNE visualization of the predominant cell populations identified from Seurat of ~13,000 heart cells collected via Drop-Seq. (Left panel) Cell-type predictions are based on established heart marker genes (literature) and gene set enrichment in the software GO-Elite (cellular biomarker database). (Right panel) DoubletDecon doublet predictions overlaid on top of the Seurat t-SNE plot, localized to the periphery of the major Seurat clusters. The dashed circle highlights endothelial-specific predicted doublets adjacent to fibroblasts.

(B and C) Secondary analysis of all Seurat-identified endothelial cells with (B) all doublets included and (C) doublets excluded with DoubletDecon prior to clustering. The left panel indicates distinct endothelial cell clusters with the doublet-enriched fibroblast cells highlighted (dashed circle), while the right panel visualizes expression of a fibroblast-specific marker.

(D) Venn diagram of DoubletDecon doublet predictions with three sepearte Seurat clustering resolutions of the entire heart dataset. The numbers of doublets identified were 1,251 (5 clusters), 1,170 (8 clusters), and 1,189 (11 clusters), with 790 (63%) in common.

that may not hold true in all applications. These assumptions include the required presence of appropriate reference cell states from which to model doublets, accurate clustering of the data, that homotypic doublets are relatively benign, and that mixed-lineage or transitional cell states will have unique gene expression. Strategies to address these concerns are further discussed in the STAR Methods. While our method performed comparably to alternative approaches, these algorithms appear to be quite complementary in identifying distinct subsets of experimentally validated doublets. Using a combination of doublet detection algorithms gives the user the ability to prioritize sensitivity or specificity, depending on the properties of the data and the research question. For many, a loss of true singlets is a reasonable trade-off for excluding unwanted contaminants. For other applications the exclusion of singlets could hinder the identification of rare cell populations or transitional states. The information in Table 2 provides users a basis for informed application of multiple doublet detection tools. Ultimately, additional optimization and improvement of these methods will enable greater precision in the characterization of cells from samples with frequent doublets in diverse single-cell platforms and studies.

STAR★METHODS

Detailed methods are provided in the online version of this paper and include the following:

- KEY RESOURCES TABLE
- LEAD CONTACT AND MATERIALS AVAILABILITY
- EXPERIMENTAL MODEL AND SUBJECT DETAILS
  - Single-Cell RNA-Sequencing
- METHOD DETAILS
  - Algorithm Description
  - Parameter Tuning and Potential Limitations
  - Evaluation Dataset Processing Parameters
  - Evaluation Parameters
- QUANTIFICATION AND STATISTICAL ANALYSIS
- DATA AND CODE AVAILABILITY

SUPPLEMENTAL INFORMATION

Supplemental Information can be found online at https://doi.org/10.1016/j.celrep.2019.09.082.

ACKNOWLEDGMENTS

We acknowledge the assistance of the Cincinnati Children's Hospital Medical Center (CCHMC) Single-Cell Genomics Core and DNA Sequencing Core. We thank Phillip Dexheimer for helpful discussions. We thank Kashish Chetal for assistance with data processing and visualization. This work was partly funded by the CCHMC Research Foundation (B.C.B. and N.S.), NIH grants R01HL122661 and R21AI35595 (H.L.G.), R01HL134312 (B.C.B.), and the Evans MDS Foundation (H.L.G. and N.S.).

AUTHOR CONTRIBUTIONS

E.A.K.D., D.J.S., H.L.G., and N.S. conceived the research, performed the data analyses, and wrote the manuscript. E.A.K.D. and P.-J.V.C. implemented the algorithm. D.J.S. provided statistical expertise and advice related to the algorithm and assistance with data processing. I.V.-A. and B.C.B. provided the heart single-cell DropSeq data. H.L.G., B.C.B., and H.S. provided critical advice and oversight related to algorithm design and evaluation.

DECLARATION OF INTERESTS

The authors declare no competing interests.

Received: July 17, 2018
Revised: July 25, 2019
Accepted: September 25, 2019
Published: November 5, 2019

REFERENCES

Butler, A., Hoffman, P., Smibert, P., Papalexi, E., and Satija, R. (2018). Integrating single-cell transcriptomic data across different conditions, technologies, and species. Nat. Biotechnol. *36*, 411–420.

Chen, H., Albergante, L., Hsu, J.Y., Lareau, C.A., Lo Bosco, G., Guan, J., Zhou, S., Gorban, A.N., Bauer, D.E., Aryee, M.J., et al. (2019). Single-cell trajectories reconstruction, exploration and mapping of omics data with STREAM. Nat. Commun. *10*, 1903.

Churko, J.M., Lee, J., Ameen, M., Gu, M., Venkatasubramanian, M., Diecke, S., Sallam, K., Im, H., Wang, G., Gold, J.D., et al. (2017). Transcriptomic and epigenomic differences in human induced pluripotent stem cells generated from six reprogramming methods. Nat. Biomed. Eng. *1*, 826–837.

DePasquale, E.A.K., Schnell, D., Dexheimer, P., Ferchen, K., Hay, S., Chetal, K., Valiente-Alandí, Í., Blaxall, B.C., Grimes, H.L., and Salomonis, N. (2019). cellHarmony: cell-level matching and holistic comparison of single-cell transcriptomes. Nucleic Acids Res. *gkz789*. https://doi.org/10.1093/nar/gkz789.

Duan, Q., McMahon, S., Anand, P., Shah, H., Thomas, S., Salunga, H.T., Huang, Y., Zhang, R., Sahadevan, A., Lemieux, M.E., et al. (2017). BET bromodomain inhibition suppresses innate inflammatory and profibrotic transcriptional networks in heart failure. Sci. Transl. Med. *9*, eaah5084.

Emig, D., Salomonis, N., Baumbach, J., Lengauer, T., Conklin, B.R., and Albrecht, M. (2010). AltAnalyze and DomainGraph: analyzing and visualizing exon expression data. Nucleic Acids Res. *38*, W755–W762.

Goldstein, L.D., Chen, Y.J., Dunne, J., Mir, A., Hubschle, H., Guillory, J., Yuan, W., Zhang, J., Stinson, J., Jaiswal, B., et al. (2017). Massively parallel nanowell-based single-cell gene expression profiling. BMC Genomics *18*, 519.

Gong, T., and Szustakowski, J.D. (2013). DeconRNASeq: a statistical framework for deconvolution of heterogeneous tissue samples based on mRNA-Seq data. Bioinformatics *29*, 1083–1085.

Hay, S.B., Ferchen, K., Chetal, K., Grimes, H.L., and Salomonis, N. (2018). The Human Cell Atlas bone marrow single-cell interactive web portal. Exp. Hematol. *68*, 51–61.

Hulin, A., Hortells, L., Gomez-Stallons, M.V., O'Donnell, A., Chetal, K., Adam, M., Lancellotti, P., Oury, C., Potter, S.S., Salomonis, N., and Yutzey, K.E. (2019). Maturation of heart valve cell populations during postnatal remodeling. Development *146*, dev173047.

Kang, H.M., Subramaniam, M., Targ, S., Nguyen, M., Maliskova, L., McCarthy, E., Wan, E., Wong, S., Byrnes, L., Lanata, C.M., et al. (2018). Multiplexed droplet single-cell RNA-sequencing using natural genetic variation. Nat. Biotechnol. *36*, 89–94.

Kashima, Y., Suzuki, A., Liu, Y., Hosokawa, M., Matsunaga, H., Shirai, M., Arikawa, K., Sugano, S., Kohno, T., Takeyama, H., et al. (2018). Combinatory use of distinct single-cell RNA-seq analytical platforms reveals the heterogeneous transcriptome response. Sci. Rep. *8*, 3482.

Langmead, B., Trapnell, C., Pop, M., and Salzberg, S.L. (2009). Ultrafast and memory-efficient alignment of short DNA sequences to the human genome. Genome Biol. *10*, R25.

Lu, Y.-C., Sanada, C., Xavier-Ferrucio, J., Wang, L., Zhang, P.-X., Grimes, H.L., Venkatasubramanian, M., Chetal, K., Aronow, B., Salomonis, N., and Krause, D.S. (2018). The Molecular Signature of Megakaryocyte-Erythroid Progenitors Reveals a Role for the Cell Cycle in Fate Specification. Cell Rep. *25*, 2083–2093.e4.

Macosko, E.Z., Basu, A., Satija, R., Nemesh, J., Shekhar, K., Goldman, M., Tirosh, I., Bialas, A.R., Kamitaki, N., Martersteck, E.M., et al. (2015). Highly Parallel Genome-wide Expression Profiling of Individual Cells Using Nanoliter Droplets. Cell *161*, 1202–1214.

Magella, B., Adam, M., Potter, A.S., Venkatasubramanian, M., Chetal, K., Hay, S.B., Salomonis, N., and Potter, S.S. (2018). Cross-platform single cell analysis of kidney development shows stromal cells express Gdnf. Dev. Biol. *434*, 36–47.

Mantsoki, A., Devailly, G., and Joshi, A. (2016). Gene expression variability in mammalian embryonic stem cells using single cell RNA-seq data. Comput. Biol. Chem. *63*, 52–61.

McGinnis, C.S., Murrow, L.M., and Gartner, Z.J. (2019). DoubletFinder: Doublet Detection in Single-Cell RNA Sequencing Data Using Artificial Nearest Neighbors. Cell Syst. *8*, 329–337.e4.

Meyer, S.E., Qin, T., Muench, D.E., Masuda, K., Venkatasubramanian, M., Orr, E., Suarez, L., Gore, S.D., Delwel, R., Paietta, E., et al. (2016). DNMT3A Haploinsufficiency Transforms FLT3ITD Myeloproliferative Disease into a Rapid, Spontaneous, and Fully Penetrant Acute Myeloid Leukemia. Cancer Discov. *6*, 501–515.

Olsson, A., Venkatasubramanian, M., Chaudhri, V.K., Aronow, B.J., Salomonis, N., Singh, H., and Grimes, H.L. (2016). Single-cell analysis of mixed-lineage states leading to a binary cell fate choice. Nature *537*, 698–702.

Satija, R., Farrell, J.A., Gennert, D., Schier, A.F., and Regev, A. (2015). Spatial reconstruction of single-cell gene expression data. Nat. Biotechnol. *33*, 495–502.

Stoeckius, M., Zheng, S., Houck-Loomis, B., Hao, S., Yeung, B.Z., Mauck, W.M., 3rd, Smibert, P., and Satija, R. (2018). Cell Hashing with barcoded antibodies enables multiplexing and doublet detection for single cell genomics. Genome Biol. *19*, 224.

Tirosh, I., Izar, B., Prakadan, S.M., Wadsworth, M.H., 2nd, Treacy, D., Trombetta, J.J., Rotem, A., Rodman, C., Lian, C., Murphy, G., et al. (2016). Dissecting the multicellular ecosystem of metastatic melanoma by single-cell RNA-seq. Science *352*, 189–196.

Velten, L., Haas, S.F., Raffel, S., Blaszkiewicz, S., Islam, S., Hennig, B.P., Hirche, C., Lutz, C., Buss, E.C., Nowak, D., et al. (2017). Human haematopoietic stem cell lineage commitment is a continuous process. Nat. Cell Biol. *19*, 271–281.

Villani, A.C., Satija, R., Reynolds, G., Sarkizova, S., Shekhar, K., Fletcher, J., Griesbeck, M., Butler, A., Zheng, S., Lazo, S., et al. (2017). Single-cell RNA-seq reveals new types of human blood dendritic cells, monocytes, and progenitors. Science *356*, eaah4573.

Wolock, S.L., Lopez, R., and Klein, A.M. (2019). Scrublet: Computational Identification of Cell Doublets in Single-Cell Transcriptomic Data. Cell Syst. *8*, 281–291.e9.

Yanez, A., Coetzee, S.G., Olsson, A., Muench, D.E., Berman, B.P., Hazelett, D.J., Salomonis, N., Grimes, H.L., and Goodridge, H.S. (2017). Granulocyte-Monocyte Progenitors and Monocyte-Dendritic Cell Progenitors Independently Produce Functionally Distinct Monocytes. Immunity *47*, 890–902.e4.

STAR★METHODS

KEY RESOURCES TABLE

REAGENT OR RESOURCE	SOURCE	IDENTIFIER
Critical Commercial Assays		
Chemegenes Drop-Seq beads	Chemgenes	CSO-2011
Droplet Generation Oil for Probes	Bio Rad	1863005
Nextera XT DNA Library Preparation Kit	Illumina	FC-131-1096
Maxima H Minus Reverse Transcriptase (200 U/μL)	ThermoFisher	EP0753
SPRIselect Reagent	Beckman Coulter	B23318
Deposited Data		
Single cell RNA-seq raw data	Synapse GEO	https://www.synapse.org/#!Synapse:syn18459941 GEO: GSE128934
Experimental Models: Organisms/Strains		
Black 6 mice (C57BL6/J)	The Jackson Laboratories	000664 |Black 6
Software and Algorithms		
DoubletDecon	This paper	https://github.com/EDePasquale/DoubletDecon
AltAnalyze	Olsson et al., 2016	http://altanalyze.org/
cellHarmony	DePasquale et al., 2019	http://altanalyze.org/
Seurat	Satija et al., 2015; Butler et al., 2018	https://satijalab.org/seurat/
DoubletFinder	McGinnis et al., 2019	https://github.com/chris-mcginnis-ucsf/DoubletFinder
Scrublet	Wolock et al., 2019	https://github.com/AllonKleinLab/scrublet
Other		
scRNA-seq UMI counts of PBMCs demultiplexed using Demuxlet	Kang et al., 2018	GEO: GSE96583
scRNA-seq UMI counts of PBMCs demultiplexed using Cell Hashing	Stoeckius et al., 2018	GEO: GSE108313
1:1 mixture of fresh frozen human (HEK293T) and mouse (NIH 3T3) cells	10X Genomics Single Cell Gene Expression Datasets	https://support.10xgenomics.com/single-cell-gene-expression/datasets/2.1.0/hgmm_12k
Single cell RNA-seq analysis of melanoma	Tirosh et al., 2016	GEO: GSE72056
Single-cell RNA-Seq for unbiased analysis of developmental hierarchies	Olsson et al., 2016	GEO: GSE70245

LEAD CONTACT AND MATERIALS AVAILABILITY

Further information and requests for resources and reagents should be directed to the Lead Contact, Nathan Salomonis (nathan.salomonis@cchmc.org). This study did not generate new unique reagents.

EXPERIMENTAL MODEL AND SUBJECT DETAILS

Single-Cell RNA-Sequencing

Transverse aortic constriction (TAC) was performed on C57BL6/J wild-type (WT) 8-10-week-old male mice (The Jackson Laboratories and confirmed via echocardiographic analysis, similar to previously described; Duan et al., 2017). Mice with an estimated pressure gradient across the aortic constriction below 40 mmHg were not included in the experiments. Hearts (n = 4, pooled) were collected at 14 days post-TAC, perfused with ice-cold PBS to remove red blood cells followed by perfusion with 50 mM KCl to arrest the heart in diastole and then fixed for 4 hours in freshly prepared 4% PFA at 4 °C, rinsed with PBS and cryoprotected in 30% sucrose/PBS overnight before embedding in OCT (Tissue-Tek). DropSeq was performed as previously described (Macosko et al., 2015). The quantity and quality of cDNA was measured using an Agilent Bioanalyzer hsDNA chip. To generate a library cDNA was fragmented and amplified (12 cycles) using the Nextera XT DNA Sample prep kit with three separate reactions of 600, 1,200 and 1,800 pg input cDNA. The libraries were pooled and purified twice using 0.7X volume of SPRIselect beads. The purified libraries were quantified using an hsDNA chip and were sequenced on an Illumina HiSeq 2500 using the sequencing parameters described

in the DropSeq protocol. Reads were aligned to the mm10 mouse genome using Bowtie2 (Langmead et al., 2009) and tagged with the gene name of the overlapped exon. Gene reads were counted by unique UMIs per cell and a digital expression matrix was created. All animal procedures were performed and approved according to the Department of Laboratory Animal Medicine and the University Committee on Animal Resources at Cincinnati Children's Hospital Medical Center.

METHOD DETAILS

Algorithm Description

Data Input

DoubletDecon accepts simple-tab-delimited cell cluster and marker gene result text files produced from the Iterative Clustering and Guide Gene Selection (ICGS) and Seurat workflows, as well as other tools when properly reformatted (Olsson et al., 2016; Satija et al., 2015). The software ICGS is a component of the easy-to-use AltAnalyze toolkit (http://www.altanalyze.org) (Emig et al., 2010). ICGS has been previously demonstrated to have excellent sensitivity to detect rare and transitional populations not identified by other approaches from scRNA-Seq data (Churko et al., 2017; Hay et al., 2018; Hulin et al., 2019; Lu et al., 2018; Magella et al., 2018; Meyer et al., 2016; Olsson et al., 2016; Yanez et al., 2017). The outputs of ICGS that are used as inputs for DoubletDecon are: 1) the clustered expression file containing only the selected discriminating genes and ordered cells, with cell cluster and gene cluster labels, 2) the groups file containing all cells with labels for cell cluster, and optionally 3) the full expression matrix containing all cells and all genes (with optional filtering for minimum number of expressed genes). The first two should be in tab-delimited text format, which is standard from ICGS, or the location and file name for these same files, while the full expression matrix must be location and file name only. Cells typically excluded as low expression outliers should be removed prior to analyses.

DoubletDecon can also accept files generated from the Seurat analysis pipeline through the built-in function Seurat_Pre_Process(). This function takes as input: 1) the normalized expression matrix or counts file that can be generated through Seurat's NormalizeData function, 2) the top discriminating (marker) gene list from Seurat's top_n function, with n selected by the user, and 3) cluster identities from the final Seurat object, which is accessed using @ident for the object. These inputs are transformed into the three ICGS-format files that can be used as input for DoubletDecon.

While ICGS and Seurat are directly supported, example input files are located in the GitHub repository associated with this project and similar inputs can be created with clustering and feature selection information from a wide variety of supervised and unsupervised methods. Please note, standard quality control methods for each scRNA-Seq platform as recommended by the manufacturer should be followed.

Processing and Optional Cell-Cycle Removal

Processing of the input data includes label standardization and optional cell-cycle gene removal. Cell-cycle removal may enable the software to better identify similar cell clusters if such gene expression profiles are present in the input dataset. All cell or gene cluster names are converted to numeric identifiers in ascending order. It should be noted that this does not change the original data but does change the cluster labels used for the outputs of DoubletDecon. If gene clusters are provided (versus simply using the top 1,000 variably expressed genes, for example) and cell-cycle gene removal is indicated, each gene cluster is examined separately for enrichment of KEGG cell-cycle gene sets using clusterProfiler package. If significant enrichment is discovered Benjamini-Hochberg adjusted $p \leq 0.05$), these genes are removed from all downstream analyses. Cell-cycle removal requires the input of species and an internet connection.

Cluster Merging

Prior to the creation of synthetic doublets, DoubletDecon attempts to join transcriptionally similar cell clusters. This step is necessary to maintain distinct cell references for subsequent deconvolution steps and can be visually evaluated and optimized in the software from the cluster merge plots and visualized heatmaps (see below). Related clusters can result from various biological and technical factors, including transcriptionally similar but distinct cell populations, patient/donor differences, or cells with few genes expressed. DoubletDecon measures similarity between cell clusters using Pearson correlation of either centroids (default) or medoids of the cell clusters. Medoids would be advisable only in cases where the frequency of doublets significantly contributes to the average gene expression profile in the cluster.

This process can be explained by the following method. Let $\{C_1, C_2, \ldots, C_K\}$ represent the cell clusters after processing the input data. Let $\{\theta_i\ i = 1{:}k\}$ denote the centroids or medoids, of the cell clusters based on the supplied marker genes. Let r_{ij} be the Pearson correlation between θ_i and θ_j. A binary correlation matrix B is derived from $\{r_{ij}\}$, with $B_{ij} = 1$ if $r_{ij} \gtrsim \rho_T$ and 0 otherwise, where

$$\rho_T = \text{mean}\{r_{ij}\} + \rho' \times \text{sd}\{r_{ij}\}\,\forall i,\ j$$

The correlation scaling parameter ρ' (rho prime) is user-defined, with a default value of 1. Lower values of ρ' will result in more clusters being combined and higher values of ρ' will retain more of the original clusters. The binary correlation matrix B is output as a heatmap to aid visual assessment. Additionally, the Shiny application enables the user to input all other parameters to generate a list of cluster merging heatmaps and associated valid ρ' values for easier selection of this parameter. If high cluster similarity is detected, i.e., at least one r_{ij} is $\gtrsim \rho_T$, Markov clustering with the mcl() function from the R package 'MCL' is used to define new clusters so that the similarity between clusters is minimized (Figure 1D). This clustering method finds the optimal number of cell clusters in the binary

correlation heatmap to represent the dataset based on overlapping similarities. By using this approach, DoubletDecon is able to effectively handle datasets with ambiguous clustering, which can result from too many cell clusters in the input dataset. Let $\{A_1, A_2, ..., A_n\}$ represent the cell clusters after the (possible) Markov clustering and $\{\alpha_i\ i = 1{:}n\}$ the corresponding centroids (medoids). These serve as reference clusters and centroids in subsequent steps.

The purpose of the next two steps in DoubletDecon is to determine if the gene expression profile of a cell is more similar to cells from an individual cell-population or synthetic doublets from two distinct cell-populations.

Synthetic Doublet Generation

Sets of synthetic doublets are generated for each of the n-choose-2 unique pairwise combination of clusters in $\{A_i; i = 1{:}n\}$, with cells randomly sampled within each cluster and the resulting gene expression averaged. For this reason, results from DoubletDecon are expected to vary slightly from run to run, based on the selection of reference cells. An option is provided to create synthetic doublets with either a 50%/50% contribution from two individual cells (default) or a weighted average for 30%/70% and 70%/30% synthetic doublets, with the former option referred to as "50-50" throughout the paper and the latter as "30-70." If this option is chosen, a total of 3 x n-choose-2 synthetic doublet sets will be created. The number of synthetic doublets created per set is user-defined (default = 100). We recommend selecting the number of synthetic doublets per cluster set based on both the number of cells in the dataset and the heterogeneity within the clusters. There should be enough synthetic cells created to represent the diversity of gene expression profiles within each cluster for best doublet identification. For datasets larger than 1000 cells, a rough estimate of the ideal number of synthetic doublets would be 10% of the total cell number. Let $\{D_i; i = 1{:}m\}$ denote the clusters of synthetic doublets and $\{\delta_i; i = 1{:}m\}$ the corresponding centroids (or medoids).

Remove Step

The "Remove" step of DoubletDecon uses deconvolution with the R package 'DeconRNASeq' and function DeconRNASeq()(Gong and Szustakowski, 2013) to estimate the relative plausibility of a cell's membership to each of the reference clusters $\{A_i; i = 1{:}n\}$(default parameters). Gong et al. proposed the use of quadratic programming to find the optimal solution to the non-negative least-squares constraint problem in microarray data, then refined the method to account for the added variation and bias in mRNA-seq data. DeconRNASeq performs deconvolution on each cell expression profile, using the reference cluster centroids $\{\alpha_i\ i = 1{:}n\}$ as the references for the deconvolution. The result, which we term a Deconvolution Cell Profile (DCP), is a vector of length n containing the percentage estimates of the contribution of each reference cell-state (cluster centroid) for queried cell (sums to 100%) (example in Figure 1C). While it seems logical that a doublet will consist of a perfect 50% contribution from each cell within the droplet, this is not always the case due to differing levels of transcriptional activity between cells and drop-out, particularly in datasets of low sequence depth. Each synthetic doublet profile in $\{\delta_i; i = 1{:}m\}$ also undergoes deconvolution with $\{\alpha_i\ i = 1{:}n\}$ as the references. Finally, the DCP of each cell in the dataset is compared to: 1) the centroid DCP for cells in each cluster in $\{A_i; i = 1{:}n\}$ and 2) the average DCP of each of the synthetic doublet cluster, using Pearson correlation for more than 2 clusters (Euclidean when the number of clusters = 2). If a cell's DCP is most strongly correlated or has the smallest distance to a synthetic doublet DCP, it is labeled as a putative doublet, with results of the "Remove" step provided in the "DRS_doublet_table" output file and the deconvolution estimates for each real cell given in the "DRS_results" output file.

The purpose of the next two steps in DoubletDecon is to determine if any of the putative doublet cells have sufficiently unique gene expression to warrant their reclassification to non-doublets.

Recluster Step

Cells that are labeled as putative doublets in the "Remove" step are reorganized into new doublet clusters in DoubletDecon's "Recluster" step, while also being removed from their original clusters. This is done by grouping together cells which have a similar DCP. Specifically, putative doublets that share the same two highest predicted contributing clusters are grouped together. In the final groups and expression files, the DCP group labels indicate the two highest correlated DeconRNASeq reference cell-types, alphabetically sorted (e.g., cluster-1 | cluster-2). If the option to include synthetic profile references with 30-70 was selected, there will be three possible group suffixes for each pair of original clusters: "even" for 50%/50%, "one" for 70%/30%, and "two" for 30%/70%, which allows for more granular recovery of non-doublet cells in the next step. We denote the doublet clusters formed in this step by $\{Z_j; j = 1{:}k\}$.

Rescue Step

In the "Rescue" step of DoubletDecon, unique gene expression present in doublet clusters is identified based on gene-by-gene comparisons. Let $\{A^*_i; i = 1{:}n\}$ denote the reference cell clusters after removal of any putative doublets. For each doublet cluster Z_j, a composite dataset $\{Z_j, A^*_i; i = 1{:}n\}$ is created and a 1-way ANOVA (factor is cluster identifier) is conducted for each gene. The p value corresponding to the n degree of freedom overall test for cluster differences and the p values for pairwise comparisons with Z_j (Tukey post hoc adjustment) are captured. If 1) the p value for the overall test is ≤ 0.05, 2) Z_j is significantly different from each cluster in $\{A^*_i; i = 1{:}n\}$ on the basis of the n Tukey-adjusted p values each being ≤ 0.05, and 3) Z_j has higher mean expression of the gene than each cluster in $\{A^*_i; i = 1{:}n\}$, then the doublet cluster Z_j is said to uniquely express that gene. This procedure is repeated for each doublet cluster $Z_j\ j = 1{:}k$.

If a doublet cluster Z_j has fewer than U unique genes identified through this process, all cells in the cluster are flagged as doublets and are written to the "Final_doublets_groups" and "Final_doublet_exp" files. If, on the other hand, a doublet cluster Z_j has $\geq$ U unique genes, the cells initially clustered as doublets (e.g., B cell | NK-cell) with a minimum number of unique genes expressed relative to the original clusters are re-assigned as singlets and reincorporated into the non-doublet expression matrix. The value of U is

user defined, with the default value of 4, which was chosen as it performed well in both the gold-standard Demuxlet peripheral blood mononuclear cell (PBMC) dataset (GEO: GSE96583) and in an evaluated mouse dataset of verified non-doublets (GEO: GSE70245). When choosing to run DoubletDecon without the "Rescue" step, the final doublets are defined as those putative doublets identified through deconvolution.

The "Rescue" step can also quickly and efficiently evaluate all genes in the expression dataset, versus only the "marker" genes, for unique gene expression regardless of the number of cells included in the input data file (suggested minimum number of unique genes for use with the full gene list is 30, which was guided by the verified mouse non-doublet evaluation dataset). Both ANOVA and Tukey post hoc tests are performed on 1,000 gene subsets, making it possible to process any file, regardless of the original size, within the memory constraints of the machine on which it's operating. Additionally, both of the above statistical tests were written to only require the total sums (TS) and sum of squares (TSS) for each cell cluster. Tukey tests were performed by comparing every doublet cluster to all original clusters using this aggregation technique to quickly calculate the means and total cells per cluster needed for the whole gene block at once. Testing was performed on a machine with 8GB of RAM and 4 cores, each processing a block of 1,000 genes (i.e., rows) for 12,000 cells (i.e., all columns) in parallel. The traditional approach would run out of memory or take > 24h to run, but using the approach above, DoubletDecon is able to perform the "Rescue" step on a test file of ~750MB containing an 22,000 × 12,000, matrix and 120 cell clusters in 2.7 minutes.

Outputs and Visualization

The intermediate and final results from DoubletDecon are optionally visualized via heatmap within the R console to help the user evaluate the exclusion of visually distinct doublet gene expression signatures. Tabular outputs of DoubletDecon include the cleaned and processed original expression and groups files, as well as separate groups and expression files for the final doublets and non-doublets. The results of the "Remove" and "Rescue" steps are also saved, as well as the deconvolution values for the synthetic doublets to assist with quality control. When running the user-interface of DoubletDecon (via Shiny), the tabular output and graphs from the command-line version are provided in an interactive environment within the application as well as saved to the given directory. Additional outputs of the Shiny application include interactive and downloadable heatmaps for initial, intermediate, and final steps of DoubletDecon, UMAP representation of original clustering with doublet overlays, and graphical displays of doublet proportion within cell clusters.

Parameter Tuning and Potential Limitations

A key determinant of the performance of DoubletDecon to detect doublets is the selection of optimal parameters. Varying these parameters can be evaluated in the DoubletDecon graphical user interface to visually assess predicted doublet cell exclusion. Notably, different datasets have different caveats requiring parameter tuning to optimize doublet removal. The following recommendations are intended to guide the user in the selection of optimal parameters for applying DoubletDecon.

Assumptions of the Approach

While this tool performs well in diverse tested datasets, it is important to note that it relies on a number of assumptions which may not hold true in all cases. First, the program assumes doublets can be identified by modeling the combination of two cell-type or cell-state expression profiles that are frequently observed in the analyzed dataset. This assumption is not true for homotypic doublets, extremely similar cell states, or when doublet cells are uniquely captured in an experiment without distinct contributing clusters. The latter can occur in rare cases when insufficient dissociation prevents isolation of a pure population of cells or where flow cytometry is used to select cells with a specific surface marker. Furthermore, although we demonstrate that transitional and mixed lineage progenitors in mouse bone marrow are defined by unique gene expression, allowing their "rescue" by DoubletDecon, this assumption may not hold in all situations. An additional caveat of our approach is the requirement of previously identified cell clusters. While, such unsupervised results could be problematic if clustering predictions are ambiguous or if cellular trajectories exist as a continuum rather than as discrete cell states, we note most all datasets have clearly defined cell populations. The use of varying synthetic proportions is among one of the most important variables in the detection of doublets with our approach (50/50 versus 30/70+50/50). Indeed, varying such proportions is important in the analysis of different dataset types, where the user may wish to conservatively remove possible doublets (e.g., global unsupervised clustering) or carefully ensure all such possible doublets are removed at the cost of some singlets (e.g., focused cell-type heterogeneity analyses). Interestingly, false positive doublet predictions were associated with cells which had lower sequencing depth, suggesting that poor quality cells are more likely to be considered multiplets due to weaker similarity to reference cell cluster centroids. Finally, DoubletDecon relies on the modeling of synthetic doublets from transcriptionally distinct cell populations, which often requires that clusters are merged, using the ρ' parameter. If clusters with highly similar transcriptomes are not merged, over-calling of doublets can occur, whereas over-joining of clusters can under-call doublets by combining distinct cell-states (see below). Importantly, if distinct doublet clusters are present, the program may not effectively identify these cells unless they are merged with similar larger clusters. Although defined ground-state truths do not exist for most datasets, as shown in the analysis of over-loaded scRNA-Seq, the reanalysis after doublet removal can be used to verify the removal of confounding doublets within a datasets (Figure 5).

Doublet Visualization

Users can interactively adjust software parameters and evaluate the success of doublet removal empirically through the graphical user interface and associated programmatic outputs (Figure S1). Likely doublets can be visually observed within a dataset in multiple ways, including visual inspection of presumed population-specific marker genes (heatmap, UMAP/PCA projection), 2) cells oriented

between clusters via low-dimensional visualization (UMAP/PCA visualization), or 3) the appearance of obvious cell-state hybrid expression profiles within a heatmap. DoubletDecon contains interactive options to determine if likely doublets are removed and adjust parameters to further monitor their exclusion or inclusion. The primary parameters to adjust in DoubletDecon are: 1) the merging of similar clusters (ρ′) for synthetic doublet creation, 2) choice of centroid or medoid for reference cluster comparison and 3) choice of 50/50 versus 30/70+50/50 synthetic doublets. Coupled with secondary unsupervised analyses (see Figure 5), users can effectively monitor the impact of doublet exclusion on datasets in which cell-types can be discerned. Note, different datasets will be subject to different variances that are likely to impact parameter selection and different parameters should be tuned accordingly.

Expression Clustering

DoubletDecon critically depends on the supplied unsupervised or supervised clustering results and basic quality control filtering (see STAR Methods, Data Input). First, we recommend applying standard quality control metrics, such as removal of cellular barcodes with few genes expressed (< 200 or 500, depending on the experiment and cell-type), log normalization, scaling, regression of artifacts and exclusion of cells with high mitochondrial content to eliminate unwanted confounding variables that impact clustering. If the user does not account for these effects, artifacts can drive the clustering or result in poor cluster homogeneity, negatively impacting doublet detection and overall interpretability. DoubletDecon provides the option to calculate reference expression profiles as centroids or medoids (centroids by default). While the choice of centroids or medoids does not typically impact doublet detection (Figure 4C), in cases were visible doublets are retained in the resulting doublet excluded heatmap, users should try the medoid option as an alternative. In such cases, the use of medoids is recommended only for datasets in which the frequency of the majority of marker genes reported is greater 50% and for data in which greater than 200 genes are expressed for cell populations. In general, including poor quality cellular barcodes will hinder doublet detection in the "Remove" step and rescue fewer singlets in the "Rescue" step, due to a lack of consistent population-specific expression. The use of centroids may not be optimal when the frequency of doublets is very high (> 25%) within a reference cluster(s), as these can result in averaged hybrid expression profiles.

Cluster Merging

The resolution of clustering performed can impact the stability of doublets predicted as shown in Figure 5D. When the resolution of clustering is high or a single cell type is analyzed, multiple similar gene-expression defined clusters will be produced (i.e., same cell lineage). If DoubletDecon is not parameterized to merge transcriptionally similar clusters, the program will attempt to create synthetic doublets between similar cell populations. Such synthetic doublets have an increased probability to look like singlets and can results in over-prediction of doublets. To deal with redundant clusters, DoubletDecon includes a cluster merging parameter called rho prime (ρ′), which varies the threshold for cluster merging prior to synthetic doublet creation. Rho prime values typically range from 0.5 to 1.5 with the default = 1 (STAR Methods, Cluster Merging). Lower values of ρ′ will result in more clusters being combined and higher values of ρ′ will retain more of the original clusters. When ρ′ is high (e.g., 1.5), no clusters from the input cluster file should be merged, whereas the maximum number of similar clusters will be joined lower ρ′ values (e.g., 0.5). The impact of inappropriately joining clusters will be a loss of transcriptional heterogeneity and loss of sensitivity to detect doublets from those distinct cell populations. Likewise, retaining transcriptionally similar clusters can result in decreased specificity. Similar problems can arise if independent observed doublet clusters (frequently occurring doublet cells that are identified as a distinct doublet cell cluster by ICGS or Seurat), are present and not effected merged in DoubletDecon with adjacent cluster(s). If not joined, such doublet clusters can reduce specificity, as synthetic doublets will be mapped to this presumably valid cell population and annotated as singlets. Where clusters are highly distinct, apply lower ρ′ values to prevent unnecessary merging of clusters. Hence, each set of clustering results used by the user should be individually examined using the Markov clustering results to select an optimized ρ′ and visually assessed in the full marker heatmap (Figures S1A and S1B). The user can select such a threshold in two ways: 1) by visually inspecting the original gene expression heatmap from ICGS or Seurat and 2) noting which clusters appear to be similar using the Markov clustering heatmap, which shows a binarized pairwise correlation plot of the gene expression centroids or medoids from each cell cluster at the selected ρ′. To assist with the selection of the ρ′ parameter, the DoubletDecon user-interface contains a "Cluster Similarity Viewer" that shows each valid Markov clustering heatmap (Figure S1B), along the spectrum of nearly all clusters merged to no cluster merged, with the associated ρ′ value. To use this option:

1. Run the DoubletDecon graphical user interface from the Shiny app (see GitHub repository).
2. Input remaining parameters and files as required.
3. Enter higher or lower value ρ' value (ρ'): "Input rhop value" option.
4. Select "Test for rho-prime values"
5. When the calculations are complete, the binarized pairwise correlation heatmaps will be displayed in the 'Cluster Similarity Viewer' tab.
6. Restart the DoubletDecon UI application and run the full workflow with the selected ρ'.

Synthetic Doublet Weighting

An important additional consideration for parameter tuning of DoubletDecon is the selection of either conservative (50/50 contribution of different cell-types) or relaxed (30/70 + 50/50 + 70/30) synthetic doublets. As noted in the Methods, DoubletDecon attempts to identify heterotypic doublets based on their Deconvolution Cell Profile (DCP) similarity to synthetic doublet DCPs and real cell DCPs. When doing so, synthetic doublets can be derived that are an equal mix of cells from two clusters (biased toward specificity) or a weighted mix, including 30%/70% and 70%/30% averages in addition to an equal mix (biased toward sensitivity). In general, the

use of 50/50 doublets is recommended when the user aims to maximize specificity over sensitivity, as this option will decrease the number of doublets predicted by restricting the diversity of possible doublets to those with an equal contribution of two transcriptomes. The use of 30/70 doublets will increase sensitivity at the cost of specificity, resulting in increased false-positive doublet calls (Figure 4C). For example, if a large number of doublets are empirically observed in a dataset (e.g., overloading of 10x Chromium port, poor tissue dissociation), the default option of 30/70 is most appropriate. Alternatively, if few cells have been captured and no clear doublet populations are observed, a more conservative (50/50) option is recommended to decrease the removal of spurious doublets, as illustrated in the example datasets shown.

Cell Cycle Exclusion

Standard unsupervised clustering methods (e.g., Seurat, ICGS) include the option to exclude predominant cell-cycle effects. When no cell cycle effects are evident in the unsupervised clustering results, this option is not needed. We recommend excluding such effects prior to analysis with DeconDecon, as cell cycle gene clusters can divide a cell population into two or more clusters that represent a single-cell type rather than doublets. As noted in the cluster merging recommendations, clusters can be merged when they are highly similar by decreasing the ρ' value. To maximize the merging of similar clusters, the cell cycle exclusion option can be set to TRUE (removeCC = TRUE) but is set to FALSE by default. When removeCC = TRUE, the software will remove cell cycle gene associated clusters through a gene set enrichment analysis procedure (Methods, Processing and Optional Cell-Cycle Removal), which will increase similarity of clusters for cluster-merging. A possible negative consequence of using this option will be the removal of cell populations almost entirely conflated with cell-cycle, such erythroblasts. Hence, this option is important to apply if cell cycle effects are evident in the unsupervised clustering results in order to merge redundant clusters. Application of this parameter can be monitored using the visualization options in the graphical user interface (Figure S1E).

Additional Considerations

Additional parameters of DoubletDecon include: 1) the selection of how many synthetics cells to generate for each cluster combination (default = 100), 2) whether to include the full expression datasets (default = FALSE) and 3) number of unique marker genes to "rescue" initial predicted clusters (default = 4 genes). While these three parameters are discussed in detailed in the above sections, we would note that all evaluations have been tested using the default parameters for these specific options and varying these parameters should not significantly impact overall results but may impact a small set of cells of interest. While including the full expression dataset for an analysis will increase runtime (depending on the size), using the full dataset can improve detection "rescue" of initial predicted doublets, especially in datasets with transitional states, by accounting for all genes not just those in the input marker file. Notably, doublet predictions will slightly vary from run-to-run, due to random synthetic doublet generation, so multiple runs may be required to determine consensus.

Evaluation Dataset Processing Parameters

The following datasets were selected for evaluation within DoubletDecon relative to established positive (known doublets) and/or negatives (known singlets). Associated doublet prediction, input data files and associated results can be obtained at https://www.synapse.org/#!Synapse:syn18460092.

Mouse-Human Mixed Dataset

Counts matrices of human and murine genes were obtained from 10X Genomics (https://support.10xgenomics.com/single-cell-gene-expression/datasets/2.1.0/hgmm_12k). This data matrix consists of cell profiles for 6,164 human, 5,915 mouse, and 741 human-mouse doublets, with consistent human gene symbols assigned to all genes, as previously described (Wolock et al., 2019).

Melanoma Biopsy Cells for Synthetic Doublet Creation

4,645 cells from a previously described scRNA-Seq dataset of 19 melanoma tumors using the SMART-Seq2 protocol were analyzed with ICGS (AltAnalyze software version 2.1.2) to predominant identify cell populations (GEO: GSE72056) (Tirosh et al., 2016). Obvious doublet cell groups within clusters were manually removed (TreeView) prior to synthetic doublet generation, leaving 4,320 cells for improved evaluation of synthetic doublets. To derive synthetic doublets for testing, the same synthetic creation pipeline used in DoubletDecon was used to create random doublets between distinct cell populations. Ten separate times, synthetic doublets were generated (n = 10% of total cells) for every pair of clusters, using the 30%–70% weighted doublets. To incorporate these synthetic cluster doublets into the dataset, we used the k-nearest neighbor scRNA-Seq alignment tool, cellHarmony (DePasquale et al., 2019).

Transitional Cell-States in Bone Marrow Progenitor Singlets

A dataset comprised of 383 hematopoietic bone marrow progenitor cells with high-confidence assigned cell-types and singlet-restricted profiles (validated via microfluidics cell capture imaging) was obtained from the GEO database along with the published ICGS unsupervised clustering results (GEO: GSE70245).

Cell Hashing Datasets

A large dataset of peripheral blood mononuclear cells (PBMC) hashed with unique cellular barcodes and isolated using 10x Chromium technology (10x Genomics) was acquired from GEO (GEO: GSE108313) (Stoeckius et al., 2018). The authors describe the run as "super-loaded" with an expected yield of 20,000 singlets and 5,000 multiplets, i.e., an overall multiplet rate of 20%. The cell doublets were identified from the cell hashing counts data in which cells from each donor were assigned a distinct hashtag oligo (HTO). A cell barcode with two or more HTOs having > 20% of the total hashtag reads was considered a doublet. For the purpose of

conducting primary analyses, 8,402 cellular barcodes with > 300 genes expressed (expressed defined as counts-per-ten-thousand $\geq$ 1) were retained. This primary dataset includes 1,869 doublets for a rate of 22.2%, consistent with the authors' expectation.

Demuxlet Datasets

A dataset containing 14,619 cells single human PBMCs isolated using a 10x Chromium instrument (10x Genomics) and sequenced using Illumina HiSeq2500 Rapid Mode with approximately 25,000 reads per cell was acquired from GEO (GEO: GSE96583) (Kang et al., 2018). The authors report an estimated doublet rate of 10.9% based on their demultiplexing procedure and note that it is consistent with the expected rate (10x technology and number of loaded cells). Following the observation that this reported rate likely estimates only the heterotypic rate (McGinnis et al., 2019; Wolock et al., 2019), we also employ an 8/7 multiplicative adjustment to arrive at an estimated overall doublet rate of 12.5%. Additionally, cell classifications of doublets and singlets were provided by the original authors, derived using Demuxlet (GEO: GSE96583). The classification for the barcodes in the dataset is as follows: 13,030 singlets, 1,565 doublets (10.7%) and 24 ambiguous. The same 300 expressed gene threshold could not be applied to the Demuxlet dataset due to too few barcodes meeting this filtering criterion. Hence, we lowered this threshold to 150 expressed genes per cell for this dataset before processing in ICGS for unsupervised cluster identification (6,525 barcodes, 1,426 doublets, 21.9% doublet rate).

The 24 barcodes with the "AMB" determination were not in the primary 6,525 barcode dataset. Additional comparative analyses among doublet-removal tools were run on the set of 14,619 barcodes–the tools applied to the full set but calculation of sensitivity and specificity excluding the 24 ambiguous barcodes (Table S1).

Mouse Heart Overloaded Dataset

A new scRNA-Seq dataset was generated from a mouse heart injury model (transverse aortic constriction) and profiled using droplet-based scRNA-Seq according to the protocol from Macosko et al. (see experimental details below) (Macosko et al., 2015). The capture was overloaded to target over 15,000 cells to identify doublets and normal cell heterogeneity in large well-defined cell populations (e.g., Endothelial). The count matrix (13,140 cells) was filtered to cells having a minimum of 200 genes expressed and 400 UMIs. Standard Seurat processing was conducted, including log-normalization, regressing out nUMI, mitochondrial proportion and cell cycle indicators (proportion of histone and Seurat G2/M transcripts), and scaling (Butler et al., 2018). PCA was conducted on the top 20% of Seurat-determined highly-variable genes. Clusters of cells were determined using the Seurat FindClusters function with 10 PCs and resolution = 0.15; a total of 8 clusters were identified and cell type of each was determined using previously identified marker genes (Seurat FindMarkers function). Results were displayed using TSNEPlot and FeaturePlot functions. The dataset and associated metadata were deposited into the open-access Synapse data sharing platform (https://www.synapse.org/#!Synapse:syn18459941).

Evaluation Parameters

DoubletDecon

ICGS was run using AltAnalyze version 2.1.2 from input read counts or normalized count matrices using the software default options(Olsson et al., 2016). For DoubletDecon, the ICGS primary output files (DataPlots/MarkerFinder or ICGS directories) were used in their native form while Seurat input files were created by applying the Seurat_Pre_Process function to the Seurat normalized expression data (converted to log2), reduced to the top 50 seurat-identified marker genes for each cluster. For all analyses, relevant parameters were set to PMF = TRUE, useFull = FALSE, only50 = FALSE (30-70), min_uniq = 3 or 4 (suggested value for useFull = FALSE), num_doubs = 100, and centroids = TRUE. Additionally, in the mouse Bone Marrow progenitor data, the parameters were also set to useFull = TRUE (along with the suggested value of min_uniq = 30) and only50 = TRUE (50-50) for testing, as indicated in the manuscript when applicable. The correlation scaling parameter ρ' (rho prime) values varied by dataset due to differing degrees of cluster similarity. Selection of values was guided by the Cluster Similarity Viewer in the Shiny version of DoubletDecon and are as follows: Cell Hashing = 1.2, Demuxlet = 1.2, Mouse/Human = 1.5 (no merging with only 2 clusters), Transitional Singlets = 1.2, Heart = 1.5, and Melanoma Synthetics = 1.3.

DoubletFinder

DoubletFinder version 2.0.1 was downloaded from https://github.com/chris-mcginnis-ucsf/DoubletFinder on May 13, 2019 and was run in the RStudio (version 1.1.447) environment for R (version 3.5.2) on a MacBook (High Sierra OS); functions for Suerat version 3 were used. We created Seurat (version 3.0.0) objects from count matrices following the example code provided on the DoubletFinder website, including log normalization, scaling, regressing out nCount_RNA, and PCA and tSNE dimension reduction.

Scrublet

Scrublet version 0.2 was downloaded from https://github.com/swolock/scrublet on May 3, 2019 and installed and run as a python 3.6.3 program on a linux computing cluster. Count matrices and gene lists were prepared and inputted following following the vignette provided on the Scrublet website.

DoubletFinder Runs on the Cell Hashing Datasets

Ten runs of DoubletFinder were conducted on each dataset, with a new seed set prior to each call. In all runs, the artificial doublet proportion pN was set to 0.25 (per authors' recommendation) and the neighborhood size parameter pK was determined using the bcvm workflow as presented in the online vignette. Following McGinnis et al. (2019), we retained 10 PCs and set the variable gene expression and dispersion thresholds at 0.025 and 0.65 respectively to select 2000 variable genes. A Seurat analysis identified 8 cell clusters in each dataset, with resulting estimated homotypic doublet proportions of 0.328 and 0.254 in the filtered dataset (8,402 barcodes) and 11,980 barcodes datasets, respectively. The doublet rate was set to 0.20 for the primary analyses (Table 1).

DoubletFinder Runs on the Demuxlet Datasets

Ten runs of DoubletFinder were conducted on each dataset, with a new seed set prior to each call. In all runs, pN was set to 0.25 and pK was determined using the bcvm workflow. Following McGinnis et al. (2019), we retained 10 PCs and set the variable gene expression and dispersion thresholds at 0.05 and 0.85 respectively to select 2000 variable genes. Consistent with the authors' report, a Seurat analysis identified 8 cell clusters in each dataset, with resulting estimated homotypic doublet proportions of 0.244 and 0.260 in the filtered dataset (6,525 barcodes) and 14,619 barcodes datasets, respectively. The doublet rate was set to 0.125 for the primary analyses (Table 1) and ranged from 0.10 to 0.219 for alternative dataset filtering options (Table S1).

Scrublet Runs on the Cell Hashing Datasets

Ten runs of Scrublet were conducted on each dataset. Following the online vignette (https://github.com/swolock/scrublet/blob/master/examples/scrublet_basics.ipynb), the following values were used for pre-processing: min_counts = 2, min_cells = 3, min_gene_variability_pctl = 85 and n_prin_comps = 30. Following Wolock et al. (2019), we set the parameters k = 50 and r = 5. For the primary analysis of the filtered dataset (8,402 barcodes), the doublet rate rho-hat was set to 0.20 and the mean-centering and variance normalization options produced a bimodal synthetic doublet score distribution and a common value of theta = 0.37 suitably separated the two distributions across all runs.

Scrublet Runs on the Demuxlet Datasets

Ten runs of Scrublet were conducted on each dataset. Following Wolock et al. (2019), we set the parameters min_counts = 2, min_cells = 3, min_gene_variability_pctl = 75, n_prin_comps = 25, k = 50 and r = 5. For the primary analysis of the filtered dataset (6,525 barcodes), the doublet rate rho-hat was set to 0.125 and the mean-centering and variance normalization options produced a bimodal synthetic doublet score distribution for all runs. Automatic estimation of the theta parameter by Scrublet was found to be reasonable based on visual inspection of all runs. For the supplemental analyses (Table S1), rho-hat was varied between 0.10 and 0.219 and the z-score and log data transformations were both run. If upon review of the synthetic doublet score histograms it was found that the automatic determination of theta was reasonable, performance was calculated on the basis of the predicted doublets. If however the automatic determination of theta did not perform well (typically an estimated theta in the extreme right tail of the distribution and/or lack of bimodality of the distribution), a consensus value of theta was determined based on inspection of all 10 histograms in a set of runs.

QUANTIFICATION AND STATISTICAL ANALYSIS

For datasets in which there is knowledge of true doublet and singlet cells (Synthetic, Mouse-Human, Cell Hashing, and Demuxlet), measurements for the performance of DoubletDecon, Scrublet and DoubletFinder are reported in terms of sensitivity and specificity. Sensitivity is calculated as the number of true doublets called by the doublet detection tool divided by the total number of known doublets. Specificity is calculated as the number of true singlets called as such by the doublet detection tool divided by the total number of known singlets in the dataset. For the Fluidigm hematopoietic progenitor dataset, with microscopy validated singlets, only specificity has been calculated. The ANOVA in the "Rescue" step is considered significant with a p value for the overall test $\leq$ 0.05 and with all Tukey post hoc adjusted pairwise p values $\leq$ 0.05.

DATA AND CODE AVAILABILITY

Melanoma (GEO: GSE72056), Bone Marrow Progenitor (GEO: GSE70245), Cell Hashing (GEO: GSE108313), and Demuxlet (GEO: GSE96583) data files were downloaded from the Gene Expression Omnibus. Mouse-Human hybrid data was downloaded from the 10x Genomics Website (https://support.10xgenomics.com/single-cell-gene-expression/datasets/2.1.0/hgmm_12k). Mouse heart cell data were generated at Cincinnati Children's Hospital Medical Center and deposited at Synapse (https://www.synapse.org/#!Synapse:syn18459941) and Gene Expression Omnibus. The accession number for the Mouse heart cell data reported in this paper is GEO: GSE128934. Accession information for the datasets used within this manuscript can be found in the Key Resources Table. DoubletDecon is provide as both a command-line R package and as a Shiny application for interactive analysis and data visualization (RStudio and desktop application for Mac). DoubletDecon is available from https://github.com/EDePasquale/DoubletDecon with a vignette on its use and optional user-defined parameters (requires R version 3.5.0 or later). The Shiny application can optionally produce the function calls to reproduce the same functions on the command-line.

Cell Reports, Volume *29*

Supplemental Information

DoubletDecon: Deconvoluting Doublets from Single-Cell RNA-Sequencing Data

Erica A.K. DePasquale, Daniel J. Schnell, Pieter-Jan Van Camp, Íñigo Valiente-Alandí, Burns C. Blaxall, H. Leighton Grimes, Harinder Singh, and Nathan Salomonis

Tool	Cell Count	ρ' (rank)	Minimum Unique	Sensitivity (Std. Dev.)	Specificity (Std. Dev.)
DoubletDecon	6,525	1.0 (1)	4†	42.66% (1.84%)	84.82% (1.47%)
DoubletDecon	6,525	1.0 (1)	5	44.73% (1.01%)	83.70% (1.07%)
DoubletDecon	6,525	1.0 (1)	6	45.40% (0.60%)	83.18% (0.38%)
DoubletDecon	6,525	1.0 (1)	10	45.52% (0.60%)	82.17% (0.52%)
DoubletDecon	6,525	1.1 (2)	4†	56.89% (1.45%)	80.98% (0.84%)
DoubletDecon	6,525	1.1 (2)	5	60.27% (0.58%)	79.98% (0.67%)
DoubletDecon	6,525	1.1 (2)	6	60.83% (0.90%)	79.31% (0.59%)
DoubletDecon	6,525	1.1 (2)	10	62.92% (0.59%)	77.49% (0.46%)
DoubletDecon	6,525	1.2 (3)	4†	57.45% (1.16%)	81.17% (0.59%)
DoubletDecon	6,525	1.2 (3)	5	60.39% (0.72%)	79.44% (0.66%)
DoubletDecon	6,525	1.2 (3)	6	60.46% (0.84%)	79.49% (0.57%)
DoubletDecon	6,525	1.2 (3)	10	62.24% (0.70%)	77.85% (0.81%)
DoubletDecon	14,619	1.1 (1)	4†	51.52% (3.10%)	83.58% (1.68%)
DoubletDecon	14,619	1.1 (1)	5	54.63% (2.20%)	82.09% (0.93%)
DoubletDecon	14,619	1.1 (1)	6	57.76% (1.02%)	80.98% (0.70%)
DoubletDecon	14,619	1.1 (1)	10	59.32% (0.33%)	79.80% (0.58%)
DoubletDecon	14,619	1.15 (2)	4†	51.11% (2.21%)	83.48% (0.88%)
DoubletDecon	14,619	1.15 (2)	5	55.37% (1.72%)	81.65% (1.15%)
DoubletDecon	14,619	1.15 (2)	6	57.40% (1.50%)	80.87% (0.91%)
DoubletDecon	14,619	1.15 (2)	10	59.35% (0.60%)	79.89% (0.52%)
DoubletDecon	14,619	1.2 (3)	4†	59.40% (1.95%)	78.02% (1.35%)
DoubletDecon	14,619	1.2 (3)	5	61.49% (1.93%)	76.61% (1.30%)
DoubletDecon	14,619	1.2 (3)	6	63.60% (1.27%)	75.41% (0.92%)
DoubletDecon	14,619	1.2 (3)	10	65.63% (0.60%)	73.92% (0.72%)

Tool	Cell Count	Rate	Homotypic Adjusted	Sensitivity (Std. Dev.)	Specificity (Std. Dev.)
DoubletFinder	6,525	10%	-	22.27% (3.05%)	93.44% (0.85%)
DoubletFinder	6,525	10%	7.6%	17.70% (2.62%)	95.28% (0.73%)
DoubletFinder	6,525	10.7%	-	23.44% (3.21%)	92.88% (0.89%)
DoubletFinder	6,525	10.7%	8.1%	18.84% (2.64%)	94.91% (0.73%)
DoubletFinder	6,525	12.5% (loading)†	-	26.44% (3.82%)	91.40% (1.07%)
DoubletFinder	6,525	12.5% (loading)†	9.5%	21.31% (2.75%)	93.87% (0.76%)
DoubletFinder	6,525	15%	-	30.54% (3.99%)	89.35% (1.12%)
DoubletFinder	6,525	15%	11.3%	24.51% (3.11%)	92.34% (0.88%)
DoubletFinder	6,525	21.9% (true)	-	39.89% (5.17%)	83.12% (1.44%)
DoubletFinder	6,525	21.9% (true)	16.6%	32.87% (3.93%)	88.02% (1.09%)
DoubletFinder	14,619	10%	-	62.57% (1.69%)	96.30% (0.19%)
DoubletFinder	14,619	10%	7.6%	48.75% (1.17%)	97.56% (0.14%)
DoubletFinder	14,619	10.7% (true)	-	65.37% (1.72%)	95.85% (0.21%)
DoubletFinder	14,619	10.7% (true)	8.1%	51.94% (1.37%)	97.36% (0.17%)
DoubletFinder	14,619	12.5% (loading)†	-	72.14% (2.01%)	94.65% (0.25%)
DoubletFinder	14,619	12.5% (loading)†	9.5%	58.87% (1.58%)	96.70% (0.19%)
DoubletFinder	14,619	15%	-	78.71% (2.38%)	92.62% (0.30%)
DoubletFinder	14,619	15%	11.3%	66.81% (1.68%)	95.55% (0.20%)
DoubletFinder	14,619	21.9%	-	87.14% (1.69%)	85.96% (0.24%)
DoubletFinder	14,619	21.9%	16.6%	80.93% (2.15%)	91.55% (0.25%)

Tool	Cell Count	Rate	Normalization (theta)	Sensitivity (Std. Dev.)	Specificity (Std. Dev.)
Scrublet	6,525	10%	Z-score (man. 0.23)†	25.67% (0.59%)	95.66% (0.13%)
Scrublet	6,525	10%	Log (man. 0.30)	17.15% (0.38%)	97.82% (0.08%)
Scrublet	6,525	10.7%	Z-score (man. 0.25)†	25.63% (0.38%)	95.72% (0.11%)
Scrublet	6,525	10.7%	Log (man. 0.30)	18.20% (0.37%)	97.60% (0.11%)
Scrublet	6,525	12.5% (loading)†	Z-score (auto.)†	31.00% (2.32%)	93.94% (1.04%)
Scrublet	6,525	12.5% (loading)†	Log (man. 0.30)	21.24% (0.41%)	96.92% (0.06%)
Scrublet	6,525	15%	Z-score (auto.)†	27.61% (2.77%)	95.12% (0.84%)
Scrublet	6,525	15%	Log (man. 0.33)	23.11% (0.35%)	96.54% (0.07%)
Scrublet	6,525	21.9% (true)	Z-score (auto.)†	32.03% (1.52%)	93.54% (0.68%)
Scrublet	6,525	21.9% (true)	Log (man. 0.42)	24.05% (0.54%)	96.33% (0.07%)
Scrublet	14,619	10%	Z-score (auto)†	59.86% (1.11%)	94.68% (0.28%)
Scrublet	14,619	10%	Log (auto)	84.92% (0.29%)	92.51% (0.17%)
Scrublet	14,619	10.7% (true)	Z-score (auto)†	58.32% (0.92%)	95.12% (0.20%)
Scrublet	14,619	10.7% (true)	Log (auto)	85.57% (0.42%)	91.91% (0.26%)
Scrublet	14,619	12.5% (loading)†	Z-score (auto)†	59.61% (2.48%)	94.78% (0.74%)
Scrublet	14,619	12.5% (loading)†	Log (auto)	87.02% (0.48%)	90.61% (0.41%)
Scrublet	14,619	15%	Z-score (auto)†	62.38% (4.88%)	93.62% (2.00%)
Scrublet	14,619	15%	Log (auto)	87.29% (1.17%)	90.04% (1.27%)
Scrublet	14,619	21.9%	Z-score (man. 0.42)†	67.81% (0.39%)	91.49% (0.28%)
Scrublet	14,619	21.9%	Log (auto)	88.92% (0.76%)	87.56% (1.48%)

Table S1. **Relative performance of doublet removal tools, Related to Table 1.** Each indicated tool was applied ten times to the Demuxlet dataset with the indicated number of cells with different cell-gene expression filtering options. ρ': cluster merging parameter for reference dataset, high rank indicating option with the least cluster merging; minimum unique: number of uniquely expressed genes in a doublet cluster necessary for cluster to be "Rescued"; rate: putative doublet rate supplied to tool; homotypic adj: adjustment applied to reduce rate to account for (undetectable) homotypic doublets; normalization: count data either z-score or log transformed; theta: doublet score threshold for classifying a cell as a doublet, user-specified value or auto(matic) determination by tool. †=default value specified in original manuscript or online documentation (when applicable). See STAR METHODS for additional details.

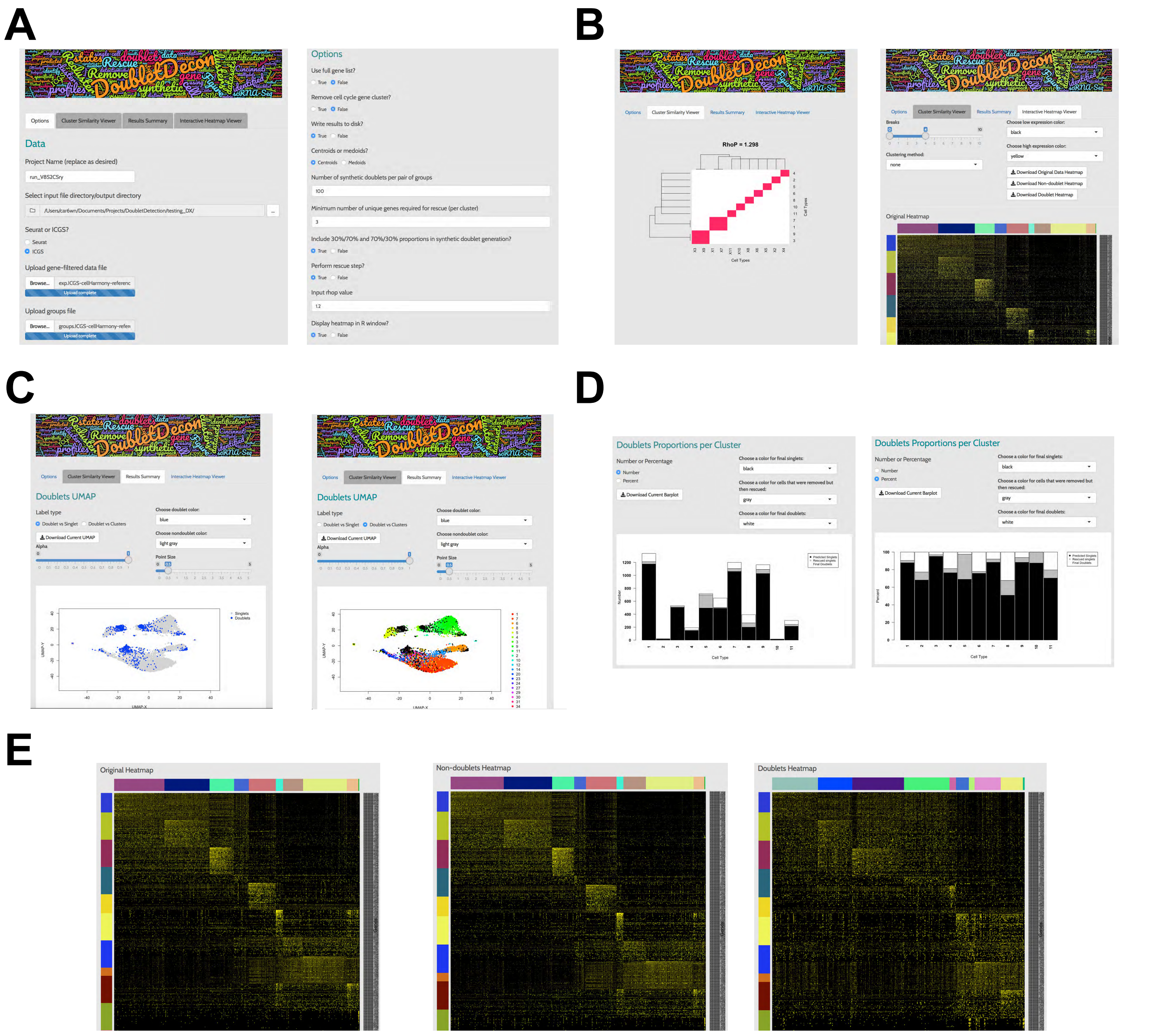

Figure S1. Interactive analysis, visualization and parameter tuning in the DoubletDecon graphical user interface, Related to the STAR Methods. Example usage of DoubletDecon is shown via the Shiny app for a PBMC dataset with cells from 8 separate donors with doublets detected by Demuxlet (Kang et al., 2018). A) Loading of input files from either ICGS or Seurat (left) and doublet detection parameters (parameters). The indicated tabs shown in the interface can be selected to adjust parameters or interactively view the data. B) Visualization of cluster similarity (centroid) to select the proper cluster merging threshold (ρ' values) to combine similar clusters prior to synthetic doublet creation (left). Here, a ρ' value 1.2-1.3 results in equivalent merging of clusters. The gene expression of markers for the original clusters are shown (right), with clusters re-labelled from x1-x11 (11 clusters). C) Visualization of the final predicted doublets (following "Rescue"), in a UMAP plot without (left) or with (right) indicated ICGS clusters. Predicted doublets typically will exist at the boundaries between clusters or at the cluster peripheries. D) Stacked bar charts of the number (left) and percentage (right) of cells in each cell cluster predicted to be doublets at the end of the end of the program, those that were initially predicted to be doublets but then rescued and singlets. E) Displayed interactive heatmap for the input scRNA-Seq cluster results prior to doublet removal (left) and after doublet removal for predicted singlets (middle) and predicted doublets (right).

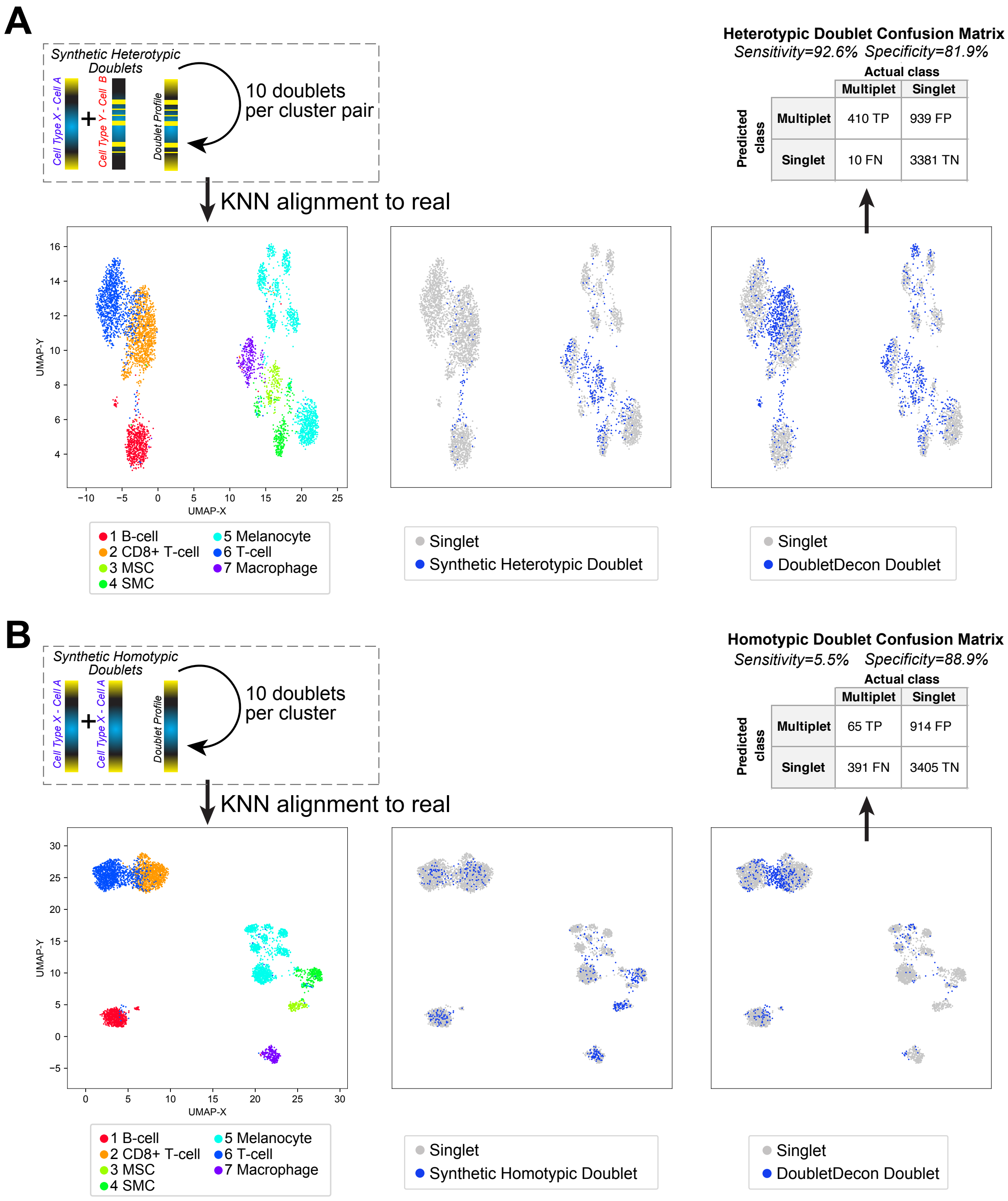

Figure S2. Detection of synthetic heterotypic doublets in human melanoma scRNA-Seq, Related to the STAR Methods. The analysis schema is shown for the evaluation of DoubletDecon on synthetic (A) heterotypic or (B) homotypic doublets of varying complexity (created with a 50/50 mix only). The same workflow used in DoubletDecon to produce synthetic doublets for doublet determination was applied in the creation of doublets for testing. Ten separately generated sets of synthetic doublets resulted in a sensitivity and specificity estimates (representative example shown in the displayed confusion matrix). UMAP plots derived from the ICGS clustering results shown for all annotated cell clusters (left), in silico derived synthetic doublets (middle) and DoubletDecon predicted doublets via deconvolution analysis (right). Labels for each cell population were independently derived through ICGS using its default cellular biomarker gene database via GO-Elite gene-set enrichment analysis.

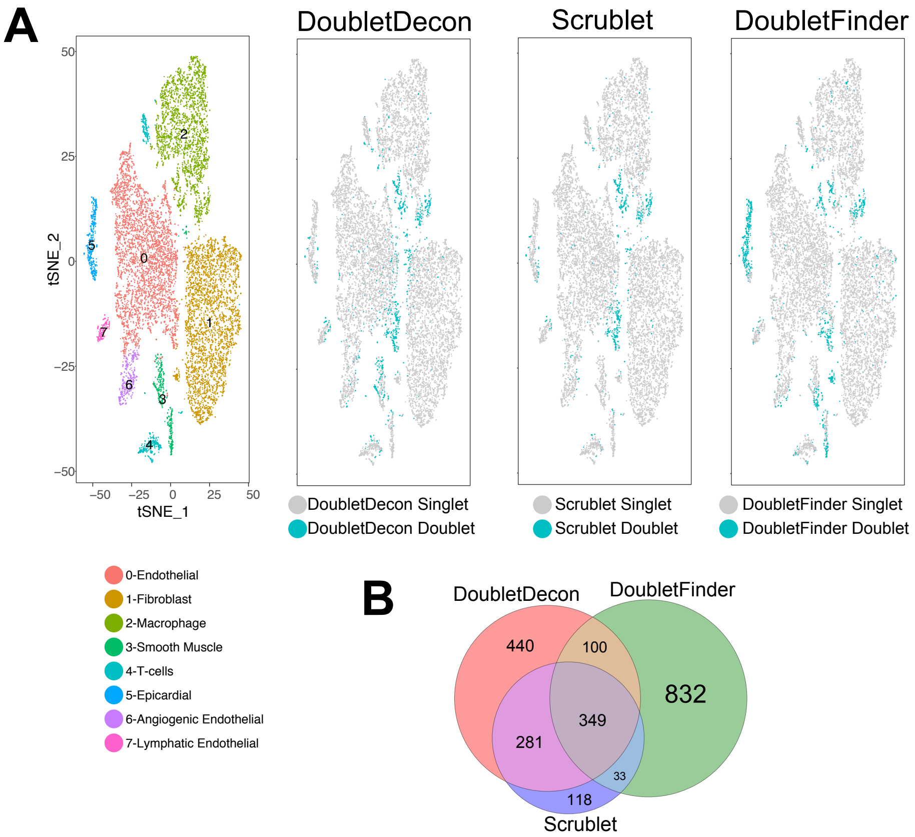

Figure S3. Common doublet cell population predictions in overloaded scRNA-Seq from mouse heart failure, Related to Figure 5. A) t-SNE visualization of the predominant cell populations identified by Seurat of ~13,000 heart cells collected via Drop-Seq. (Bottom) Labels for each cell population were independently derived through ICGS using its built-in cellular biomarker database gene-set enrichment analysis (GO-Elite function). Projected predicted doublets are shown for the three evaluated algorithms on projected into the t-SNE plot. B) Overlaps in doublet predictions for the three methods, indicating distinct overlapping subsets by all three algorithms. The parameters for Scrublet and DoubletDecon were according to the authors' recommendations or optimized for stability across runs.
